# A Genetic Screen for Pathogenicity Genes in the Hemibiotrophic Fungus *Colletotrichum higginsianum* Identifies the Plasma Membrane Proton Pump Pma2 Required for Host Penetration

**DOI:** 10.1371/journal.pone.0125960

**Published:** 2015-05-19

**Authors:** Martin Korn, Johannes Schmidpeter, Marlis Dahl, Susanne Müller, Lars M. Voll, Christian Koch

**Affiliations:** Department of Biology, Division of Biochemistry, Friedrich-Alexander University Erlangen-Nuremberg, Staudtstrasse 5, 91058 Erlangen, Germany; University of Nebraska-Lincoln, UNITED STATES

## Abstract

We used insertional mutagenesis by *Agrobacterium tumefaciens* mediated transformation (ATMT) to isolate pathogenicity mutants of *Colletotrichum higginsianum*. From a collection of 7200 insertion mutants we isolated 75 mutants with reduced symptoms. 19 of these were affected in host penetration, while 17 were affected in later stages of infection, like switching to necrotrophic growth. For 16 mutants the location of T-DNA insertions could be identified by PCR. A potential plasma membrane H^+^-ATPase Pma2 was targeted in five independent insertion mutants. We genetically inactivated the Ku80 component of the non-homologous end-joining pathway in *C*. *higginsianum* to establish an efficient gene knockout protocol. *Chpma2* deletion mutants generated by homologous recombination in the *ΔChku80* background form fully melanized appressoria but entirely fail to penetrate the host tissue and are non-pathogenic. The *ChPMA2* gene is induced upon appressoria formation and infection of *A*. *thaliana*. Pma2 activity is not important for vegetative growth of saprophytically growing mycelium, since the mutant shows no growth penalty under these conditions. *Colletotrichum higginsianum* codes for a closely related gene (*ChPMA1*), which is highly expressed under most growth conditions. *ChPMA1* is more similar to the homologous yeast genes for plasma membrane pumps. We propose that expression of a specific proton pump early during infection may be common to many appressoria forming fungal pathogens as we found *ChPMA2* orthologs in several plant pathogenic fungi.

## Introduction

Many aspects of fungal pathology have been discovered by forward genetic screens using different strategies for generating mutant alleles. Traditionally, transposon mutagenesis and REMI mutagenesis have been used to generate mutants [1]. Since the finding that T-DNA transfer can be used for the efficient transformation of filamentous fungi [2], insertion mutagenesis by *Agrobacterium tumefaciens* mediated transformation (ATMT) has been used in many systems [3, 4]. Plant pathogens of the genus *Colletotrichum* and *Magnaporthe* have been subjected to genetic screens aimed at identifying genes involved in virulence of the pathogen [4–8]. As the genomes of both the host and the pathogen are available, the *Colletotrichum higginsianum / Arabidopsis thaliana* pathosystem is well suited for a molecular analysis of pathogenicity. *Colletotrichum higginsianum* belongs to a large genus of plant pathogenic fungi whose host spectrum includes economically important crop plants like maize, tropical fruits and *Brassicaceae* [9]. Despite being very closely related, *Colletotrichum* species differ substantially with regard to the extent of the biotrophic infection stage [10]. Biotrophic hyphae spread to adjacent cells in the maize pathogen *Colletotrichum graminicola* and in *C*. *lindemuthianum* [11], whereas in *Colletotrichum higginsianum* biotrophy is restricted to the first infected cell [9]. After forming an appressorium, *C*. *higginsianum* penetrates the plant cell with the support of a large turgor pressure generated in the melanized appressorium. Appressoria differentiation is initiated upon physical contact with the plant cuticle and involves poorly characterized external signals that may include wax components on the plant surface. Large bulbous biotrophic hyphae are formed in the first infected cell. Necrotrophy is initiated when secondary filamentous hyphae develop that invade neighboring cells. Primary hyphae grow biotrophically between the plasma membrane of the host cell and the plant cell wall generating structures referred to as interfacial bodies which are thought to be critical areas for effector delivery [12, 13]. During this initial infection stage, the host cell remains alive and consequently host defense mechanisms and / or pathogen recognition must be suppressed. Plant pathogenic fungi use a series of different mechanisms to accomplish this. These include active suppression of defense by apoplastic and cytoplasmic effectors and mechanisms to reduce recognition of pathogen-associated molecular patterns (PAMPS) [13]. Secretion of chitin binding proteins like the LysM domain proteins Ecp6 [14] and Slp1 [15] or inhibition of host proteases [16] are common mechanisms. Early upon infection, a large number of potential effector genes are induced in *C*. *higginsianum* [12, 17]. In addition, nutrient transporters involved in phosphate uptake, nitrogen assimilation as well as drug efflux systems are upregulated during infection. Many of these membrane transporters belong to the major-facilitator-superfamily and use proton symport for uptake [18]. After the initial biotrophic phase, the fungus spreads to neighboring cells and establishes the necrotrophic stage, where host cells are actively killed [19]. During necrotrophy, carbohydrate-active enzymes, proteases and necrosis inducing peptides are upregulated [17].

Candidate pathogenicity genes can be identified on the basis of their expression pattern [12, 20], protein signatures [21] or their similarity to genes with known functions [22]. While genetic screens based on heterologous overexpression of effector genes have been successful in oomycetes and bacteria, screening for loss of function mutants with altered virulence represents the most unbiased approach to identify novel functions involved in pathogenicity. Here, we report the results of a forward genetic screen for *Colletotrichum higginsianum* genes involved in pathogenicity using ATMT. We found mutants affected in several steps of the infection process with little overlap to previous screens in *Colletotrichum* [6–8]. We identified the T-DNA insertion sites for 16 strains in the mutant collection. Furthermore, we verified the effect of 4 candidates on virulence by targeted knockout of the corresponding gene using a *ΔChku80* mutant, which increases efficiency of homologous recombination. Interestingly, we isolated five mutant alleles of a novel gene encoding a virulence-associated P-type H^+^-ATPase with a special role in host cell penetration.

## Materials and Methods

### Strains and media

*Agrobacterium tumefaciens* strain AGL1 (AGLO *recA*::*bla* pTiBo542ΔT Mop+ CbR [23], strain BAA-101 in ATCC collection, a gift of Jörg Kämper) was used for ATMT. *Agrobacteria* were transformed with plasmid DNA as described [24]. Depending on the plasmid used, transformants were selected with kanamycin (75 μg/ml) or spectinomycin (100 μg/ml). *E*. *coli* strain DH5α was used for plasmid DNA isolation and cloning. *Colletotrichum higginsianum* strains MAFF 305970 and MAFF 305635 [9] were obtained from the Ministry of Agriculture, Forest and Fisheries collection (Japan). Strain CY5535 (this study) was a single conidial isolate of MAFF 305635 and was used for insertion mutagenesis and as the parental strain for all further strain constructions. Strain CY6021 (*ΔChku80*::*nat*) (this study) was derived from CY5535 and used for targeted mutagenesis.

*C*. *higginsianum* was propagated at 25–28°C on PDA plates or in modified liquid Mathur’s medium. For induction of conidiation, strains were grown on oatmeal plates (OMA) for 7 days at 25°C in an incubator illuminated for 12 h/day. Modified Mathur’s medium [25] contained 2.8 g glucose, 1.5 g peptone, 0.5 g yeast extract. 1.2 g Mg_2_SO_4_ x 7 H_2_O and 2.7 g KH_2_PO_4_ per liter. For the preparation of solid medium, 30 g/l agar were added before autoclaving. Potato dextrose agar (PDA) was obtained from Carl Roth (Karlsruhe, Germany). OMA plates were prepared from oat flakes (organic quality, Bioland, Germany) ground in a blade coffee grinder. 3 g agar and 12.5 g oatmeal were mixed with 250 ml H_2_O in a 1 liter Erlenmeyer flask and autoclaved for 45 min. Czapek Dox medium (Sigma Aldrich) was used as a minimal medium for *C*. *higginsianum*. Auxotrophy was analyzed by supplementing minimal Czapek Dox medium with adenine (55 mg/l), arginine (55 mg/l) or lysine (76 mg/l), respectively. YEB medium (5 g/l beef extract, 1 g/l yeast extract, 5 g/l peptone, 5 g/l sucrose, 0.5 g/l MgCl_2_) and YEB plates (YEB medium containing 1.5% agar) were used for *Agrobacterium* strains. *E*. *coli* strains were propagated in LB medium according to Green and Sambrook [26]. Glycerol stocks of *C*. *higginsianum* strains were prepared by freezing conidial suspensions in 15% (w/w) glycerol at -80°C. *Arabidopsis thaliana* plants (Col-0) were grown for 5 weeks prior to infection in a growth chamber under 12 h light (22°C) / 12 h dark (19°C) cycles (91 μE/m^2^s; 70% humidity).

### Pathogenicity screening

Random insertional mutants were grown as described before, rinsed off from oatmeal plates with water and adjusted to a titer of 10^5^ conidia per ml. *A*. *thaliana* Col-0 plants were grown for five weeks in multiwell trays. Three leaves of different plants were used for droplet inoculation (4 times 5 μl droplets per leave) for each mutant strain. Pathogenicity symptoms were assessed macroscopically after six days of incubation in growth chambers. Mutants with reduced or no symptom development were tested two more times in separate experiments. Mutants showing reproducible pathogenicity defects were used for spray infection of *A*. *thaliana* Col-0 plants with a titer of 10^6^ conidia per ml. Leaves were harvested after three and four days for macro- and microscopic analysis.

### Vector and plasmid constructions

Plasmid pPK2 [27] conferring hygromycin resistance was used for the generation of insertion mutants. Plasmid pPN (S1 Text) is a pPK2 derivative conferring nourseothricin resistance (*nat*). The *Streptomyces noursei nat* coding region was from pMF1-N (S1 Text). Plasmid pCK2831 (S1 Text) was used for targeted gene replacement of *ChKU80* with the nourseothricin resistance gene. The pOSCAR system [28] was used for gene replacements with the hygromycin resistance cassette (S1 Text). Further plasmid constructions are described in S1 Text.

### RT-PCR analysis

1 μg of total RNA was used to synthesize first strand cDNA in 25 μl using RevertAid H minus reverse transcriptase (200 U), oligo (dT)_18_ primer (0.25 μg), and RiboLock RNase Inhibitor (40 U) (Thermo Fisher Scientific) according to the manufacturer’s instructions. Samples were stored at -20°C until use. cDNA was amplified in 50 μl reactions using 1 μl first strand cDNA, dNTPs (200 μM each), Taq polymerase (2.5 U) and gene specific primers (200 nM each). Routinely, 30 reaction cycles were performed. Quantitative real-time RT-PCR was performed in a MX300 real-time PCR instrument (Stratagene) using 1 μl cDNA, gene specific primers (200 nM each) and Brilliant II SYBR Green QPCR mastermix (Agilent Technologies). Primer efficiencies were calculated with the standard curve approach using the MxPro software (Stratagene). Transcript levels were measured in three technical replicates from 3 biological replicates each and standardized based on the transcript level of α-tubulin (CH063_01222) according to Pfaffl [29]. Primers used for quantitative real-time PCR are listed in S1 Text.

### Standard techniques

DNA manipulations, PCR reactions Southern blotting and plasmid DNA isolations followed standard protocols as described [26]. Southern blots were hybridized with PCR fragments randomly labeled with α- ^32^P dCTP.

### Transformation of *C*. *higginsianum* and generation of insertion mutants

*Agrobacterium tumefaciens* mediated transformation (ATMT) was used for generation of *C*. *higginsianum* transformants. The transformation protocol followed methods established by de Groot et al. [2] and techniques described in [30]. *A*. *tumefaciens* strain AGL1 harboring the binary vector pPK2 [27] or GFP expressing derivatives of pPK2 (S1 Text) were used. The *hph* gene of pPK2 is controlled by the *A*. *nidulans gpd* promoter. For ATMT, *C*. *higginsianum* conidia were grown on OMA plates for 8 days at 25°C and subsequently kept at 4°C for 2 days, which facilitates rinsing off the conidia. Conidia were rinsed off OMA plates with MM medium [31] containing 2.5% sucrose and adjusted to a titer of 10^6^/ml. *A*. *tumefaciens* strains harboring binary vectors were grown in MM medium with antibiotics at 28°C for two days and subsequently diluted to an OD_600_ of 0.15 in induction medium (IM) [31] carefully adjusted to pH 5.6 with 2-(*N*-morpholino)ethanesulfonic acid (MES) and containing 0.2 mM acetosyringone. After incubation for 6 h at 28°C, 0.1 ml bacterial cells were mixed with 0.1 ml conidial suspension (10^6^/ml). The mixtures were evenly spread onto nylon filters (mesh size 70 μm) (SEFAR NITEX 03-70/33, Sefar Group, Germany) that were placed on IM plates (IM medium with 1.5% agar). *A*. *tumefaciens* and *C*. *higginsianum* cells were co-cultivated for 2 days at 28°C. Subsequently, nylon filters were transferred to PDA plates supplemented with 80 μg/ml hygromycin, 50 μg/ml spectinomycin and 50 μg/ml cefotaxime and incubated for 2 d at 25°C (12 h light/day) to select for fungal transformants and against bacterial cells. After a second transfer to selective PDA plates, hygromycin resistant colonies became visible after 2–4 days. Usually 30 to 100 transformants per plate were obtained. Conidia from individual transformants were single colony purified on PDA plates containing hygromycin (80 μg/ml). Single colonies were further propagated for 7 days (25°C, 12 h light/dark cycle) on multiwell OMA plates (24 wells). Conidial suspensions were frozen and stored in glycerol (15% w/v) at -80°C in microtiter plates.

### Infection of Arabidopsis plants and pathogenicity assay

Five weeks old *A*. *thaliana* plants were used for spray or droplet inoculation with *C*. *higginsianum*. Deionized water was used to rinse off conidia from oatmeal agar plates that were incubated for at least seven days at 25°C and one day at 4°C. The conidia concentration was measured using a CASY1 cell counter model TT from Schaerfe Systems (Innovatis AG, Reutlingen, Germany). For spray infection, the conidial suspension was adjusted to 1 x 10^6^ conidia per ml and evenly sprayed on the plants. For droplet inoculation, 5 μl droplets (1 x 10^5^/ml) were spotted on attached leaves. To achieve high humidity conditions, infected plants were watered and placed in airtight and wetted plant trays. Infected plants were incubated in phytochambers for three to six days. Samples for DAB (3,3'-diaminobenzidine) and aniline blue staining were harvested at 3 dpi, while samples for trypan blue staining were taken 4 days post infection (S1 Text). Macroscopic symptom development was assessed after 4 (spray inoculation) or 6 days (droplet inoculation) according to lesion size and frequency.

### *In vitro* appressoria formation, penetration and turgor pressure assays

Tissue culture dishes (60 x 15 mm; Sarstedt AG; Germany) were coated with 1,16-hexadecanediol (1 ml solution, 0.1 mM in methanol), which is inductive for appressorium formation in *M*. *grisea* [32]. Though not essential, this led to more reliable results across different charges of plastic plates. After drying, 5 ml of conidia suspension (2 x 10^5^ conidia/ml) were added and incubated for 24 h at 25°C. To check the ability to penetrate a cellophane surface, sterilized dialysis tubes (Visking 36/32, Carl Roth, Karlsruhe, Germany) were put on 1.2% agarose-covered glass slides and incubated with 1 x 10^5^ conidia for two days at 25°C and high humidity conditions. The incipient cytorrhysis assay of appressoria was performed in coated tissue culture dishes with 1 x 10^6^ conidia/dish. The water was substituted after 24 h with polyethylene glycol 6000 solutions with concentrations varying from 100 to 500 mg/ml which corresponds to external turgor pressures from 70 Pa to 5.8 MPa [33]. The percentage of collapsed appressoria was quantified after 10 min of incubation in polyethylene glycol solution in three biological replicates with at least 100 appressoria each.

### Microscopy

All histochemical samples were analyzed with a Leica DMR HC microscope (Leica Microsystems, Wetzlar, Germany). Confocal laser scanning microscopy was performed using a Leica TCS SP5 II (Leica Microsystems, Wetzlar, Germany) with an HCX PL APO lambda blue 63.0 x 1.20 Water UV lens. GFP was excited with the 488 nm band of an argon laser and was detected at 496 to 556 nm. A 561 nm DPSS laser was used for the excitation of mCherry and its emission was detected between 569 and 637 nm.

### Sequence analyses and accessions

*C*. *higginsianum* DNA sequences [17] were obtained from the *Colletotrichum* Sequencing Project, Broad Institute of Harvard and MIT (http://www.broadinstitute.org/). Where indicated, the *Colletotrichum higginsianum* Genome Browser at the Max-Planck-Institute for Plant Breeding Research was used to obtain additional DNA sequences (http://gbrowse.mpiz-koeln.mpg.de/cgi-bin/gbrowse/colletotrichum_higginsianum_public/). Sequence alignments were performed with BLAST and ClustalW algorithms as implemented in the Geneious software package (www.geneious.com). *ChTRPC* (Accession KP180422) encodes a 765 aa tri-functional protein containing indole-3-glycerol-phosphate synthase and anthranilate synthase activities (yeast TRP3) followed by phosphoribosylanthranilate isomerase activity (yeast TRP1) (on contig 07584 and plasmid pCK2321 (Yeplac181 clone)). *ChPMA2* (Accession KP180423). *ChPMA1* (Accession KP261085).

## Results

### A mutant screen for genes involved in pathogenicity in *Colletotrichum higginsianum*

**Fig 1:**
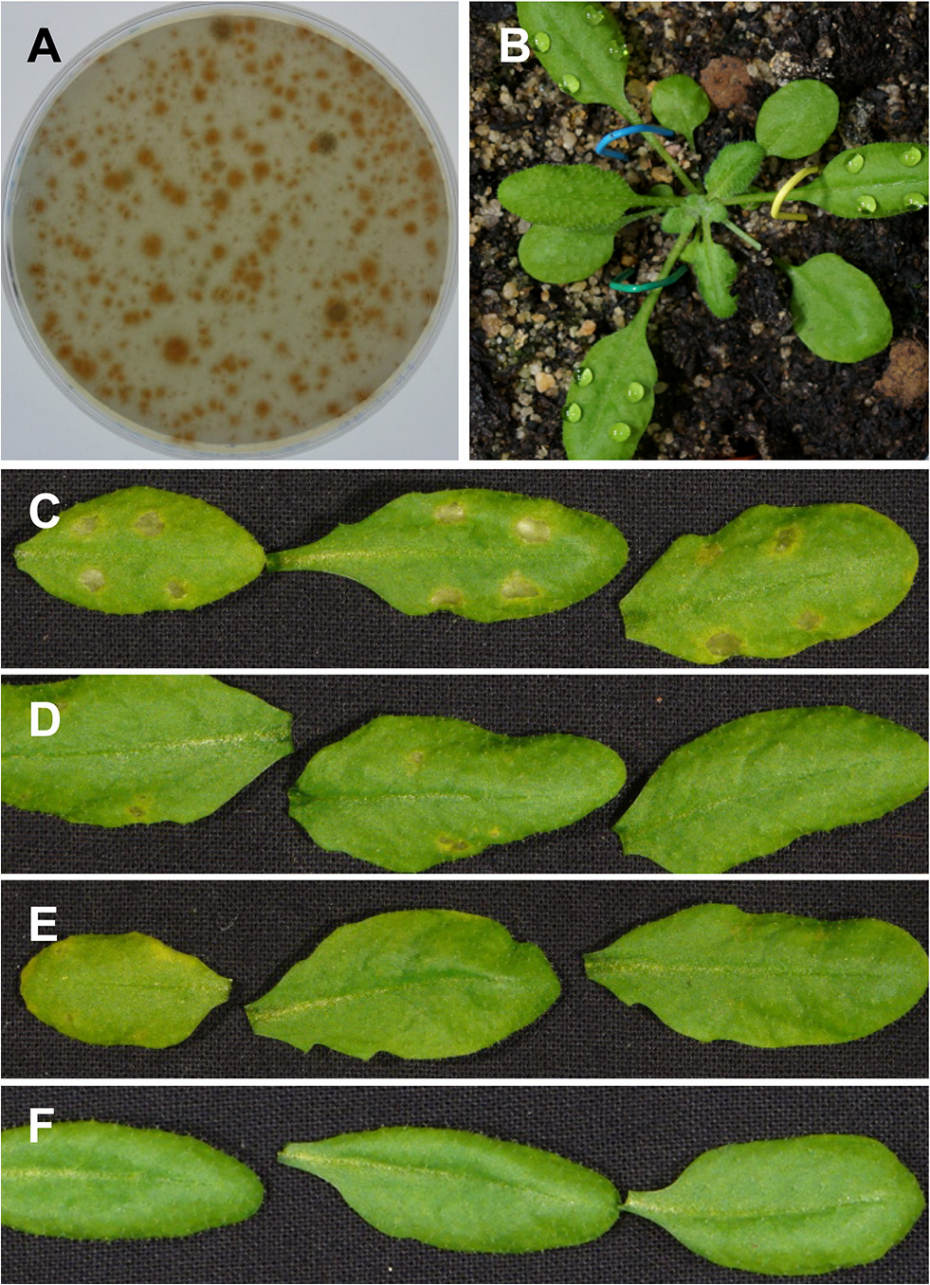
Pathogenicity screening of *C*. *higginsianum* T-DNA insertion mutants. (A) Selective PDA hygromycin plate six days after *Agrobacterium tumefaciens*-mediated transformation (ATMT) of *C*. *higginsianum* with pPK2. (B) *Arabidopsis thaliana* Col-0 plant directly after droplet inoculation with *C*. *higginsianum*. (C, D, E) Typical symptom development on Col-0 leaves 6 days after droplet infection (500 conidia/droplet) with wild type *C*. *higginsianum* strain CY5535 (C) and T-DNA insertion mutants showing reduced pathogenicity (D) or no symptoms (E). (F) Mock inoculation with water.

In order to obtain mutants affected in pathogenicity we used *Agrobacterium tumefaciens* mediated transformation (ATMT) of *C*. *higginsianum* conidia. In short, conidia were co-cultivated with agrobacteria harboring binary vectors encoding a hygromycin resistance gene in the T-DNA and were induced for T-DNA transfer with acetosyringone. Transformants were selected on PDA plates containing hygromycin (Fig 1A), single colony purified on selective plates and further propagated on OMA plates. We took care to isolate independent transformants by picking a minority of transformants per plate to avoid biases towards certain growth characteristics of the transformants. Using this protocol, we isolated about 7200 independent transformants that were kept as frozen stocks before testing their virulence on plants. All transformants were tested by drop inoculating attached leaves with a defined titer of 500 conidia/drop (Fig 1B), which allowed screening a large number of transformants per experiment under controlled conditions. To screen for insertion mutants with altered virulence, transformants showing no or reduced symptoms after 6 days of infection (Fig 1C–1F) were selected and rescreened twice under the same conditions to exclude false negatives from the virulence screen. Of the 7227 transformants initially screened, 559 candidates showed reduced or no symptoms in the first screen. For 186 of the candidates the phenotype was reproduced in the second screen. 75 mutants, i.e. 1% of the initially analyzed transformants, showed an effect on pathogenicity after three rounds of droplet inoculation and were considered potential pathogenicity or virulence mutants. We refer to these mutants as *vir* mutants. Detailed phenotypic analysis was performed using spray inoculation of whole plants with high titers of 10^6^ conidia per ml (S1 Fig). Progression of disease symptoms using droplet inoculation was slightly delayed relative to spray inoculation. After spray infection, the parental strain formed appressoria on leaves within 8–12 h. Shortly thereafter, penetration of the host cuticle was detectable in microscopic pictures of leaves stained with trypan blue. After 2 days, primary biotrophic hyphae were detectable underneath >50% of appressoria. After 3 days, symptoms started to become visible. After 4–6 days strong necrotic lesions were macroscopically evident (S1 Fig) as described [8, 9, 34].

### Phenotypic analysis of *C*. *higginsianum* pathogenicity mutants

In order to characterize the phenotypes of the *C*. *higginsianum* mutants created by random insertional mutagenesis, the formation of primary hyphae or secondary hyphae *in planta* was analyzed. In addition, the ability of the mutants to grow on minimal medium and to form appressoria *in vitro* was quantified. Furthermore, callose papilla deposition and H_2_O_2_ accumulation [35, 36] beneath appressoria was analyzed in order to determine altered host defense (Table 1). Since all strains were propagated as conidial suspensions, mutants with defects in conidiation though potentially interesting would not have been identified. Mutants with reduced virulence are expected to be either defective in suppressing host defense mechanisms or to lack functions directly required for plant penetration or *in planta* growth [15]. Insertion mutants showing a general slow growth phenotype may also lead to reduced symptoms. Among the mutants recovered, only nine mutants exhibited a slow growth phenotype on agar plates showing that for most pathogenicity mutants reduced virulence is not due to slow growth. We isolated three auxotrophic mutants, of which two turned out to be adenine and arginine auxotrophs, respectively. Adenine auxotrophs are expected to exhibit a short phenotypic lag due to the immediate requirement of adenine during DNA synthesis, while arginine biosynthesis was reported to be necessary for penetration and invasive growth of *C*. *higginsianum* [37]. Based on the ability to develop infection structures, the 75 *vir* mutants could be assigned to four groups. Group I consisted of mutants with reduced (<10% of conidia) or absent appressoria formation *in vitro*. Group II mutants were not capable to penetrate the host or to form primary hyphae while still being able to form at least some appressoria (>10% of conidia). Group III mutants penetrated and formed at least some primary hyphae (>10% of appressoria) but were clearly impaired in the subsequent formation of secondary hyphae and in the establishment of necrotrophic growth. The remaining pathogenicity mutants could not be placed into these categories and formed Group IV.

**Table 1:** Phenotypes of *C*. *higginsianum* pathogenicity mutants.

Mutant	VS^1^	AP^2^	PH^3^	SH^4^	Callose^5^	ROS^6^	MM^7^
WT	+++	90%	39% ^a^	3% ^a^	11%	7%	100%
**Group I: Mutants impaired in appressoria formation (<10% of conidia)**							
*vir-1*	0	0	0	0	0	-	++
vir-2*	0	0	0	0	0	0	0
*vir-3*	0	0	0	0	n.d.	n.d.	+
*vir-4*	0	0	0	0	0	-	+
*vir-5*	0	0	0	0	n.d.	n.d.	+
**Group II: Mutants impaired in penetration or establishment of primary hyphae (AP formation >10%, PH formation ≤1%)**							
vir-10*	+	+++	0	0	-	-	++
vir-11*	0	++	0	0	n.d.	0	+
vir-12*	0	+++	0	0	-	0	++
*vir-13*	+	++	0	0	-	+	0
*vir-14*	0	++	0	0	-	0	++
vir-15*	0	+++	0	0	-	0	++
*vir-16*	0	+++	0	0	0	0	+
*vir-17*	0	+++	0	0	0	0	++
*vir-18*	0	+	0	0	0	0	++
*vir-19*	0	+++	0	0	-	0	++
*vir-20*	0	+++	0	0	-	0	++
*vir-21*	0	+++	0	0	-	0	++
*vir-22*	0	+++	0	0	-	-	++
*vir-23*	+	++	0	0	-	-	++
*vir-24*	0	+++	0	0	-	0	++
*vir-25*	+	+++	0	0	-	-	++
*vir-26*	0	+++	0	0	-	0	++
*vir-27*	0	+++	0	0	0	-	++
*vir-28*	0	+++	0	0	n.d.	0	++
**Group III: Switch mutants (PH formation >10%, SH formation ≤1%)**							
vir-40*	0	++	++	0	-	-	++
vir-41*	+	+++	++	0	-	-	++
vir-42*	+	+++	++	0	0	++	++
vir-43*	0	+++	++	0	-	0	0
vir-44*	+	+++	++	0	-	0	++
vir-45*	+	+++	++	0	-	0	++
*vir-46*	+	+++	++	0	-	+	++
*vir-47*	+	+++	++	0	-	++	++
*vir-48*	+	+++	++	0	+	++	++
*vir-49*	+	+++	++	0	+	++	+
*vir-50*	+	+++	++	0	-	-	++
*vir-51*	+	+++	++	0	0	-	++
*vir-52*	+	+++	++	0	-	-	++
*vir-53*	0	+	++	0	0	++	++
*vir-54*	+	+++	+	0	-	0	++
*vir-55*	+	+++	++	0	0	-	++
*vir-56*	0	+	++	0	0	-	++
**Group IV: Not classified**							
vir-70*	0	+++	+	+++	-	-	++
vir-71*	+	+++	+++	++	-	-	++
*vir-72*	+	++	+	0	-	++	++
vir-73*	0	+++	+	0	0	-	++
vir-74*	+	+++	++	+	-	+	++
*vir-75*	+	+++	+	0	-	++	++
*vir-76*	+	+++	+++	+	0	-	++
vir-77*	++	+++	+++	+++	-	++	++
*vir-78*	+	+++	+++	+	-	0	++
vir-79*	+	+++	+	+	-	-	++
*vir-80*	+	+++	++	+	-	+	++
*vir-81*	+	++	++	++	0	-	++
*vir-82*	++	+++	+++	+++	-	-	++
*vir-83*	+	+++	++	+++	-	++	++
*vir-84*	+	+++	++	+++	-	-	++
*vir-85*	+	+++	++	+	-	0	++
*vir-86*	+	+++	+	+	-	0	++
*vir-87*	++	+++	+++	+++	-	+	++
*vir-88*	+	+++	++	+	-	0	++
*vir-89*	+	+++	+	+	-	0	++
*vir-90*	+	+++	++	++	-	0	++
*vir-91*	+	+++	+	++	0	-	++
*vir-92*	++	+++	+++	+++	-	-	++
*vir-93*	++	+++	+++	+++	-	-	++
*vir-94*	++	+++	+++	+++	-	++	++
*vir-95*	+	+++	++	+	-	0	++
*vir-96*	+	+++	+	+	-	-	++
*vir-97*	+	+++	+	+	-	-	++
*vir-98*	0	+++	+	0	-	-	++
*vir-99*	+	+++	++	+	+	++	++
*vir-100*	+	+++	++	+	-	++	++
*vir-101*	0	+++	++	+	-	++	++
*vir-102*	++	+++	+++	WT	-	-	++
*vir-103*	0	++	+	0	-	-	++

* Mutant was generated by transformation with a plasmid other than pPK2. See Text S1.

^a^ Wild type data is given 3 days after infection since strong symptom development after 4 days prevented reliable quantification. After 4 days, the number of secondary hyphae ranged from >30 to >60%.

^1^ Visible symptoms after 4 days of spray infection; 0: no visible symptoms. +: reduced symptoms, ++: slightly reduced symptoms, +++: WT.

^2^ *in vitro* appressoria formation on 1,16-hexadecanediol-coated petri dishes in percentage of conidia; 0: <10%, +: 10–24%, ++: 25–50%, +++: >50%.

^3^ Formation of primary hyphae 4 days after spray-infection of *A*. *thaliana* Col-0 in percentage of appressoria; 0: <1%, +: 1–10%, ++: 11–25%, +++: >25%.

^4^ Formation of secondary hyphae 4 days after spray-infection of *A*. *thaliana* Col-0 in percentage of primary hyphae; 0: ≤ 1%, +: 1–10%, ++: 11–20%, +++: >20%.

^5^ Callose papilla (stained with aniline blue) formed beneath appressoria 3 days after spray-infection of *A*. *thaliana* Col-0 in percentage of appressoria; 0: <1%,-:1–10%, +: 11–12%, ++: >12%, n.d.: not detectable.

^6^ ROS accumulating cells (stained with 3,3-diaminobenzidine) beneath appressoria 3 days after spray-infection of *A*. *thaliana* Col-0 in percentage of total appressoria; 0: <1%,-: 1–5%, +: 6–7%, ++: >7%, n.d.: not detectable.

^7^ Colony diameter on Czapek-Dox minimal medium after 5 days compared to WT; 0: no growth, +: <30%, ++: >30%.

#### Mutants impaired in in vitro appressoria formation (Group I)

**Fig 2:**
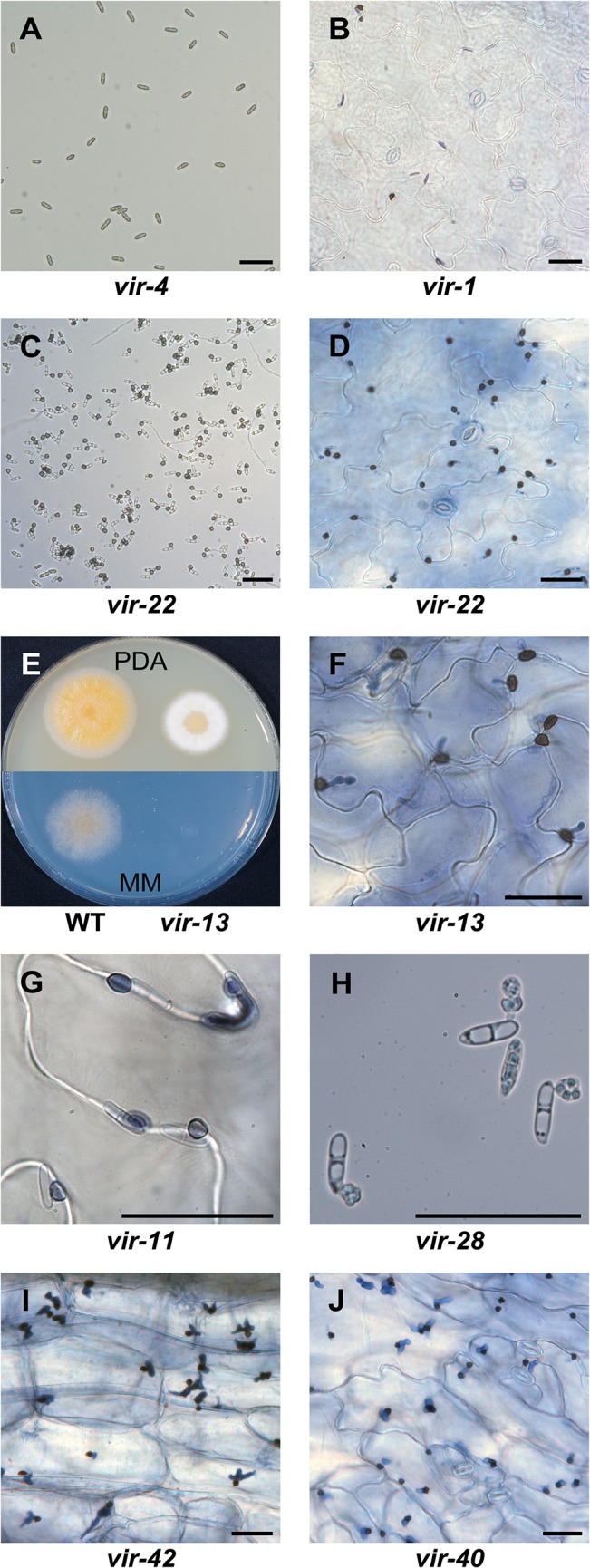
Phenotypes of selected *vir* mutants. (A) Failure of group I mutant *vir-4* to form appressoria on 1,16-hexadecanediol coated petri dishes. (B) *vir-1* mutant on trypan blue stained *A*. *thaliana* leaf four days after infection. (C) Appressoria of group II mutant *vir-22* on coated petri dishes. (D) *vir-22* on trypan blue stained leaves four days after infection. (E) Growth of *vir-13* on potato dextrose agar (PDA, after four days) and Czapek Dox plates (MM, after six days) compared to wild type *C*. *higginsianum*. (F) Trypan blue stained *A*. *thaliana* leaves four days after infection with *vir-13*. (G) Trypan blue stained *A*. *thaliana* leaves four days after infection with *vir-11*. (H) Appressoria formation of *vir-28* on coated petri dishes. (I, J) Formation of primary hyphae on trypan blue stained *A*. *thaliana* leaves four days after infection with two group III mutants (I: *vir-42*; J: *vir-40*). Scale bar = 25 μm.

Three out of five mutants of this group showed reduced (*vir-3* and *vir-5*) or impaired (*vir-2*) growth on minimal medium but normal growth on rich medium. In contrast to *vir-1* and *vir-2*, which still were able to form appressoria to some extent (4% and 6% respectively), *vir-3*, *vir-4* and *vir-5* never germinated (Fig 2A). While *vir-3* and *vir-5* also failed to form appressoria on *A*. *thaliana* leaves, the other mutants of this group developed appressoria on leaves (Fig 2B). This suggests that *vir-1*, *vir-2* and *vir-4* may be affected in sensing hydrophobic surfaces and that the plant cuticle contains additional signals which may compensate for this defect.

#### Penetration mutants (Group II)

Most of the 19 mutants in this group showed normal growth on minimal medium and *in vitro* appressoria formation at wild type level (Fig 2C) but failed to penetrate and form primary hyphae (Fig 2D). Additionally, these mutants induced less host defense responses than the wild type (Table 1). This suggests that successful penetration or formation of primary hyphae correlates with efficient pathogen recognition by the host. *Vir-13* deviates from this pattern. Even though it shows arginine auxotrophy and mildly decreased growth rate on rich medium (Fig 2E), this mutant was able to penetrate the host cuticle. Instead of bulbous primary hyphae, this mutant developed deformed infection structures (Fig 2F) similar to previously reported arginine auxotrophic *path* mutants [37]. Furthermore, this mutant induced host ROS (5.4%) and callose response (8.5%) on wild type level (6.7% ROS and 11.2% callose deposition), which was not typical for this mutant class, further indicating the necessity of penetration for strong defense responses. *Vir-11* grew slower than wild type both on minimal and rich medium and produced less melanized *in vitro* appressoria than wild type. *In planta* appressoria of this mutant were less pigmented than wild type appressoria and seemed to get stained by trypan blue, which is not usually observed (Fig 2G). *Vir-28* is the only mutant in our screen that exhibited complete loss of melanization in appressoria both *in vitro* and *in planta* (Fig 2H).

#### Mutants affected in switching to necrotrophic growth (Group III)

Mutants of this group showed appressoria formation, penetration and biotrophic growth but failed to efficiently form necrotrophic hyphae (Fig 2I and 2J). The majority of the 17 members of this group elicited less plant defense reactions than wild type, except for *vir-47*, *vir-48* and *vir-49*, which showed increased callose deposition and ROS accumulation. *Vir-42* and *vir-53* induced more ROS than wild type while no callose papillae were observed. Interestingly, mutant *vir*-*43* was able to form primary hyphae, although it did not grow on minimal media (data not shown). This behavior was unique among all *vir* mutants.

The remaining 34 mutants showed diverse phenotypes and did not show the characteristic defects of mutants from group I to III.

### A large number of mutants harbor more than one T-DNA insertion

It was reported that stable T-DNA integration by ATMT into fungal genomes is random but not necessarily restricted to single integration events [2, 27]. The number of fungal ATMT transformants containing single T-DNA copies reported from different screens ranged from 16% in *C*. *graminicola* [38] to 72% in *C*. *acutatum* [5]. Huser *et al*. [8] reported that 58% of their *C*. *higginsianum* random insertional mutants had single T-DNA insertions, while 72% of those with more than one copy had all T-DNA copies integrated at the same locus. A similar screen in *C*. *higginsianum* [7] resulted in 48% single T-DNA insertions. Since multiple T-DNA insertions and especially multiple independent insertion sites complicate the characterization of transformants, we analyzed the spectrum of T-DNA insertions by probing Southern blots of genomic mutant DNA with radiolabeled hygromycin DNA fragments (S2 Fig). Genomic DNA was digested with either SalI or BamHI. Both enzymes have single recognition sites in the transforming T-DNA. A single T-DNA insert should therefore generate one hybridizing band per insert. The number of hybridizing fragments varied between 1 and 4 (S2 Fig). Based on this analysis, 22 of the 70 mutants analyzed carried a single T-DNA insertion, 28 had two insertions and 20 had three or more insertions. A significant number of mutants (approximately 20) may contain tandem T-DNA insertions. These either showed bands with the size of a complete T-DNA (4590 bp; e.g. S2 Fig, *vir-99*) indicative of a head-to-tail arrangement, or different number of bands counts in both digests (e.g. *vir-18*, S2 Fig) suggesting a head-to-head or tail-to-tail arrangement [39, 40]. In summary, 31% of the mutants analyzed by Southern blotting could be assigned to a single T-DNA insertion event. We found no evidence that different plasmids, transformation protocols or selection conditions affected the number of insertions obtained (data not shown). However, we did not analyze whether or not the hyper-virulence of the *Agrobacterium* strain AGL1 [23] was responsible for multiple insertions.

### Identification of T-DNA insertions sites in *vir* mutants

In an ongoing effort, we used Genome Walker PCR techniques [41] to amplify genomic DNA sequences linked to T-DNA borders from 16 of the isolated virulence mutants. After ligating DNA adaptors to genomic mutant DNA digested with different blunt-cutting restriction enzymes, genomic sequences adjacent to T-DNA were PCR amplified using primers specific to adaptor sequences together with primers either specific to the left border or the right border of the inserted T-DNA. After DNA sequencing, we were able to identify genomic location of 18 T-DNA insertions. In 16 cases, only one of the two border sequences could be identified although primers specific for both the LB and the RB sequences of the integrating T-DNA were used. In one (*vir-88*) of the two mutants where we could isolate both T-DNA flanking regions, we observed a small deletion of 13 nucleotides of genomic DNA (data not shown). In three mutants (*vir-52*, *vir-53* and *vir-84*) we found tandem insertions having left border sequences fused to right border sequences. Sequences flanking the T-DNA were used as queries in nucleotide-BLAST searches against the *C*. *higginsianum* Genome Database (*Colletotrichum* Sequencing Project, Broad Institute of Harvard and MIT, http://www.broadinstitute.org/) (Table 2). We found DNA insertions inside coding regions or in potential regulatory sequences. Of 16 *vir* mutants, eight mutants (*vir-12*, *vir-14*, *vir-52*, *vir-56*, *vir-84*, *vir-88*, *vir-97* and *vir-102*) contained T-DNA sequences inserted upstream of predicted genes. For five mutants (*vir-14*, *vir-22*, vir-*24*, *vir-27* and *vir-76*), the insertion site was located inside of predicted open reading frames, while four mutants (*vir-2*, vir-*10*, *vir-51* and *vir-53*) contained T-DNA sequence downstream of annotated, potential genes. In two mutants, two separate insertion sites were identified (*vir-14* and *vir-27*). The following section describes *vir* mutants for which T-DNA insertion sites could be identified.

**Table 2:** T-DNA insertion sites in *C*. *higginsianum* ATMT mutants.

mutant	insertions^1^	T-DNA Insertion Site^2^	potential T-DNA-tagged Gene^3^	gene name	best BLASTP hit (NCBI accession)^4^	E-value, identity
*vir-2*	1	supercontig_1.2671, 583, RB	CH063_08186: 546 bp downstream	*ChADE2*	phosphoribosylaminoimidazole carboxylase (EFQ26499.1), *C*. *graminicola*	0.0, 97%
*vir-10*	2	contig05930, 16777, LB	CH063_03425: 527 bp downstream	*ChKEL2*	kelch domain-containing protein (EFQ26610.1), *C*. *graminicola*	0.0, 90%
*vir-12*	2	supercontig_1.3174, 1154, LB	CH063_09060: 154 bp upstream	*ChPMA2*	plasma-membrane proton-efflux P-type ATPase (EFQ27159.1), *C*. *graminicola*	0.0, 97%
*vir-14*	2	supercontig_1.6150, 870, RB	CH063_13013: ORF		ABC transporter (EFQ25092.1), *C*. *graminicola*	0.0, 95%
		supercontig_1.903, 6335, LB	CH063_03980: 1767 bp upstream		nucleoside-diphosphate-sugar epimerase (ENH82360.1), *C*. *orbiculare*	0.0, 85%
*vir-22*	1	supercontig_1.3174, 1748, RB	CH063_09060: ORF	*ChPMA2*	plasma-membrane proton-efflux P-type ATPase (EFQ27159.1), *C*. *graminicola*	0.0, 97%
*vir-24*	1	supercontig_1.3174, 1422, RB	CH063_09060: ORF	*ChPMA2*	plasma-membrane proton-efflux P-type ATPase (EFQ27159.1), *C*. *graminicola*	0.0, 97%
*vir-27*	2	supercontig_1.6150, 873, RB	CH063_13013: ORF		ABC transporter (EFQ25092.1), *C*. *graminicola*	0.0, 95%
		supercontig_1.826, 7944, RB	CH063_03776: ORF	*ChSTE12*	STE like transcription factor (EFQ27157.1), *C*. *graminicola*	0.0, 95%
*vir-51*	1	supercontig_1.1848, 6585, LB	CH063_06511: 1036 bp downstream		-	-
*vir-52*	2, tandem	contig00557, 11896, LB + RB	a. CH063_02404: 1221 bp upstream; b. CH063_12090: 230 bp downstream	*ChLYS1* (a)	a. alanine dehydrogenase/PNT domain-containing protein (EFQ25467.1), *C*. *graminicola*; b. FAD dependent oxidoreductase superfamily protein (XP_007280006), *C*. *gloeosporioides*	a. 4e-167, 96%; b. 7e-157, 84%
*vir-53*	2, tandem	supercontig_1.6692, RB	CH063_13555: 434 bp downstream		-	-
*vir-56*	3	supercontig_1.66, 3878, LB	CH063_00495: 76 bp upstream		peroxisomal membrane protein 24 (EFQ28871.1), *C*. *graminicola*	3e-160, 98%
*vir-76*	2	supercontig_1.56, 17248, LB	CH063_00433: ORF	*ChMAD1*	spindle assembly checkpoint component MAD1 (EFQ32105.1), *C*. *graminicola*	0.0, 90%
*vir-84*	2, tandem	supercontig_1.3742, 1175, LB	CH063_09976: 325 bp upstream	*ChRMD1*	sporulation protein RMD1 (ELA35952.1), *C*. *gloeosporioides*	0.0, 92%
*vir-88*	2, tandem	supercontig_1.5277, 868–879, RB + RB	CH063_12012: 161 bp upstream	*ChMOB2*	Mob1/phocein family protein (EFQ26211.1), *C*. *graminicola*	0.0, 99%
*vir-97*	2	supercontig_1.3174, 812, LB	CH063_09060: 496 bp upstream	*ChPMA2*	plasma-membrane proton-efflux P-type ATPase (EFQ27159.1), *C*. *graminicola*	0.0, 97%
*vir-102*	1	supercontig_1.3174, 793, LB	CH063_09060: 515 bp upstream	*ChPMA2*	plasma-membrane proton-efflux P-type ATPase (EFQ27159.1), *C*. *graminicola*	0.0, 97%

^1^ Number of T-DNAs insertions as determined by Southern Blot analysis.

^2^ Position of the T-DNA border sequence on *Colletotrichum* Database Supercontigs (*Colletotrichum* Sequencing Project, Broad Institute of Harvard and MIT, http://www.broadinstitute.org/) or on *Colletotrichum higginsianum* Database Contigs (Max Planck Institute for Plant Breeding Research, http://gbrowse.mpiz-koeln.mpg.de/cgi-bin/gbrowse/colletotrichum_higginsianum_public/). The sequenced border sequence is given as left border (LB) or right border (RB).

^3^ Gene IDs from *Colletotrichum* Databases (see 2); upstream: distance to start codon, downstream: distance to stop codon, ORF: in open reading frame.

^4^ Best BLAST hit against NCBI non-redundant protein sequences database (hypothetical and *C*. *higginsianum* proteins excluded).

***Vir-2*:** The T-DNA insertion of *vir-2* was located about 500 bp downstream of a predicted gene with high similarity to *S*. *cerevisiae ADE2* (46% amino acid identity) required for purine nucleotide synthesis. *Vir-2* showed dark pigmentation when propagated on OMA plates (S7C Fig). Increased pigmentation is a typical feature of yeast *ade2* mutants. *Vir-2* was further found to be adenine auxotrophic (Table 1). It is therefore very likely that the T-DNA insertion affects *ChADE2* expression.

**Fig 3:**
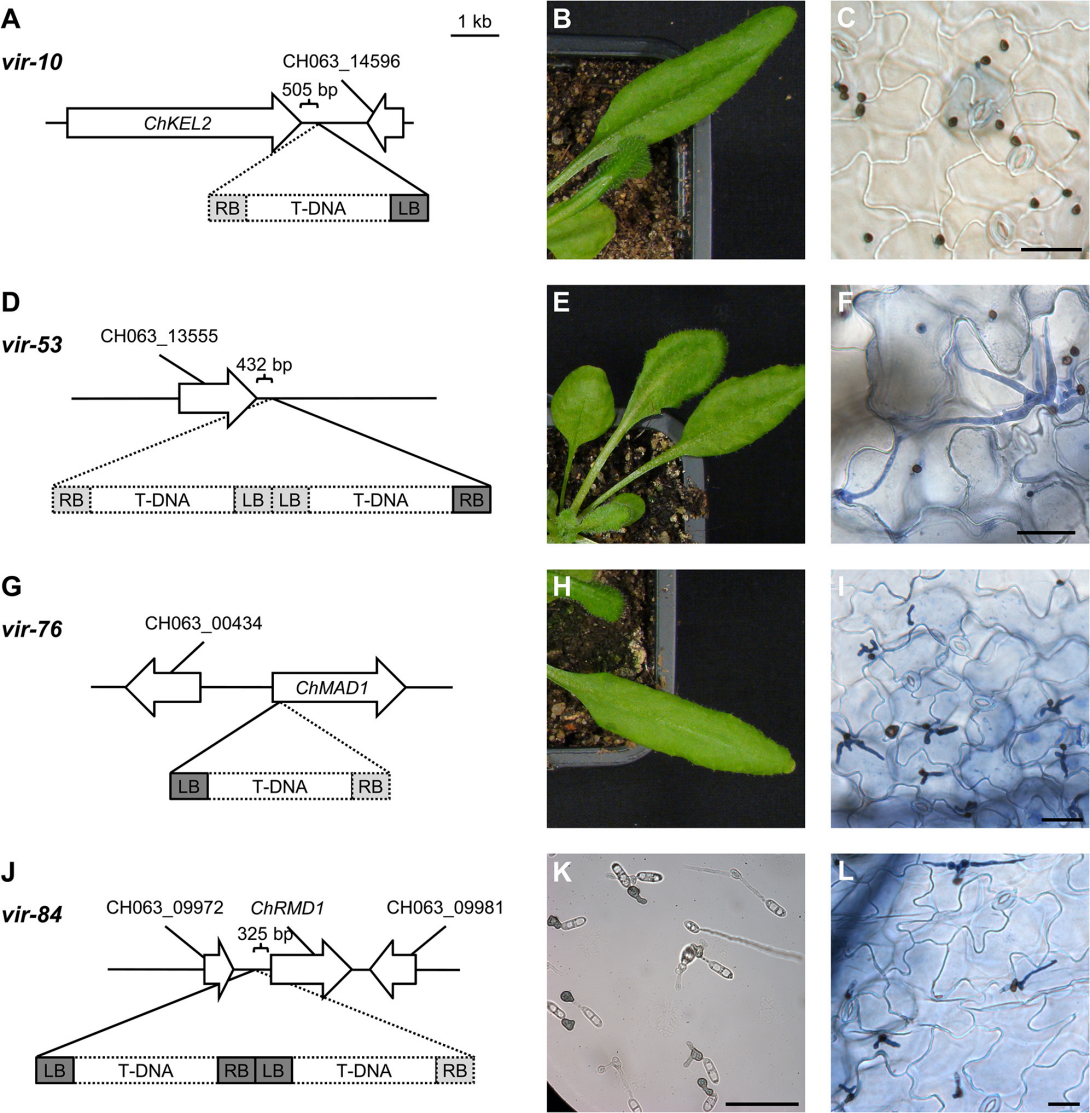
T-DNA insertion sites of selective *vir* mutants. Location of T-DNA-insertions and typical phenotypes of *vir-10* (A, B, C), *vir-53* (D, E, F), *vir-76* (G, H, I), and *vir-84* (J, K, L). T-DNA sequences isolated by Genome Walker PCR are illustrated as solid rectangles. Predicted parts of the T-DNA that were not directly sequenced are shown as dotted lines. Genes are named corresponding to Table 2 or by their Gene ID (*Colletotrichum* Sequencing Project, Broad Institute of Harvard and MIT, http://www.broadinstitute.org/). Typical phenotypes of the respective *vir* mutants shown as light microscopic images of trypan blue stained leaves 4 days after infection (C, F, I, L) and the corresponding macroscopic symptom development (B, E, H). (K) Appressoria formation of *vir-84* after 15 h on coated petri dishes. Scale bar = 25 μm.

***Vir-10*:** One of the two T-DNA insertions in the genome of this mutant is located downstream of the predicted kelch-repeat containing protein CH063_03425, which was therefore named *ChKEL2* (Fig 3A–3C). Proteins with kelch repeats were originally identified as the product of cell polarity genes in *S*. *pombe* [42]. Their yeast homologs Kel1 and Kel2 [43] are also involved in cell polarity. Interestingly, the *C*. *orbiculare* kelch repeat encoding genes *ClaKEL2* (80% amino acid identity to ChKel2) [44] and *Cokel1* [45] were identified as pathogenicity factors.

***Vir-14*:** This mutant harbors two T-DNA insertions. One is located in the ORF of a putative ABC transporter (CH063_13013) homologous to yeast ATM1, which is involved in mitochondrial transport [46]. The second insertion is upstream of a gene with unknown function (CH063_03980).

***Vir-27*:** Mutant *vir-27* has two independent T-DNA insertions. One is located at CH063_13013 and the other one is located in the ORF for a gene highly similar to the *C*. *orbiculare Ste12-*like transcription factor Cst1 (92% amino acid identity). Cst1 was shown to be required for appressorium penetration [47] and binds to PRE sequences [48]. The appressorium penetration phenotype of *vir-27* is likely to be caused by mutation of *ChSTE12*, but could also be affected by its second insertion.

***Vir-51*:** The single T-DNA insertion of this mutant was found downstream of a predicted gene (CH063_06511) without conserved domains but is conserved in *C*. *graminicola*. The protein encoded by this gene is predicted to be unconventionally secreted using SecretomeP [49].

***Vir-52*:** This mutant harbors two T-DNAs. PCR analysis suggested them to be located in a head-to-tail order downstream of a putative oxidoreductase (CH063_12090) and upstream of the predicted gene CH063_02404 with homology to yeast *LYS1*.

***Vir-53***: This mutant (Fig 3D–3F) contains a tandem T-DNA insertion 434 bp downstream of a predicted gene (CH063_13555) of unknown function with no homologs except in other *Colletotrichum* species.

***Vir-56*:** Southern blot analysis of the mutant indicated the presence of tandem insertions of multiple T-DNAs. One insertion site could be identified 76 bp upstream of a predicted open reading frame (CH063_00495) which encodes a protein containing a protein motif found in mitochondrial membrane and peroxisomal proteins (IPR003397).

***Vir-76*:** This strain produces normal appressoria but exhibits reduced pathogenicity largely due to its inability to efficiently form secondary hyphae (Fig 3G–3I). *Vir-76* harbors a T-DNA insertion in the 5’-end of a predicted open reading frame similar to the yeast spindle checkpoint gene *MAD1* [50].

***Vir-84*:** Mutant *vir-84* exhibits a unique phenotype. It produces appressoria with defects in shape, melanization and polarization (Fig 3J–3L). 18% of these appressoria were able to penetrate and form primary hyphae (Fig 3L). Secondary hyphae were observed for less than 6% of all appressoria. A tandem T-DNA insertion could be identified 325 bp upstream of a predicted gene (CH063_09976) encoding a protein with high similarity (49% identity, E-value of 9e^-130^) to the yeast sporulation protein Rmd1 [51].

***Vir-88*:** Mutant *vir-88* shows reduced symptoms upon infection. The genome of this mutant contains two T-DNAs 161 bp upstream of a predicted gene that encodes a protein similar to yeast Mob2, which is part of the RAM-pathway. This pathway is involved in regulation of cytokinesis and polarized cell growth in yeast [52] and in virulence in *Cryptococcus neoformans* and *Candida albicans* [53, 54].

### Five *vir* mutants carry insertions in *ChPMA2* encoding a potential plasma membrane proton pump

We found 5 independent mutants (*vir-12*, *vir-22*, *vir-24*, *vir-97* and *vir-102*) that carry T-DNA insertions at the locus for gene CH063_09060 coding for a predicted P-Type ATPase, which is related to the yeast plasma membrane H^+^-ATPases Pma1p and Pma2p. We call this gene *ChPMA2*. *ChPMA2* also shares sequence similarity to the previously described *LmPMA1* from *Leptosphaeria maculans* [55], which was shown to be required for pathogenicity against oilseed rape. Importantly, all 5 mutations were independent as each insertion is located at a different position of *ChPMA2* or its 5´-UTR (S3A Fig). The transcription start site is not known but there are 2 possible AUG start codons separated by only 15 nucleotides from which we tentatively assigned the first to be the translational start (+1). The T-DNA insertion sites of *vir-22* and *vir-24* are located in the open reading frame of *ChPMA2* (*vir-24*: +130; *vir-22*: +454). These mutants were characterized as class II mutants (Table 1). Both showed *in vitro* and *in planta* appressoria formation at wild type levels but completely failed to form primary or secondary hyphae even six days after spray inoculation (S3A Fig). Appressoria of *vir-22* and *vir-24* also induced less callose papillae formation (ca. 1%) compared to wild type (11%). In addition, the production of reactive oxygen species (ROS) stained with DAB was reduced in these mutants. The third potential *Chpma2* mutant v*ir-12* harbors two T-DNA insertions. One could be located 139 bp upstream of *ChPMA2*, while the other insertion site is yet unknown. The phenotype of *vir-12* (S3B Fig) is similar to mutants *vir-22* and *vir-24*. This may indicate that the insertion inactivates essential promoter elements. *Vir-102*, on the other hand, harbors a single T-DNA insertion 502 bp upstream of the potential start codon and is able to form primary and secondary hyphae, but shows reduced symptom development (S4B Fig). The insertion site found in *vir-97* is only 20 bp upstream from the insertion in *vir-102* but showed a more severe phenotype with no visible symptoms (S3B Fig). However, *vir-97* contains a second unidentified T-DNA insertion which may additionally affect pathogenicity.

### Construction of a *ΔChku80* strain deficient in non-homologous end-joining

**Fig 4:**
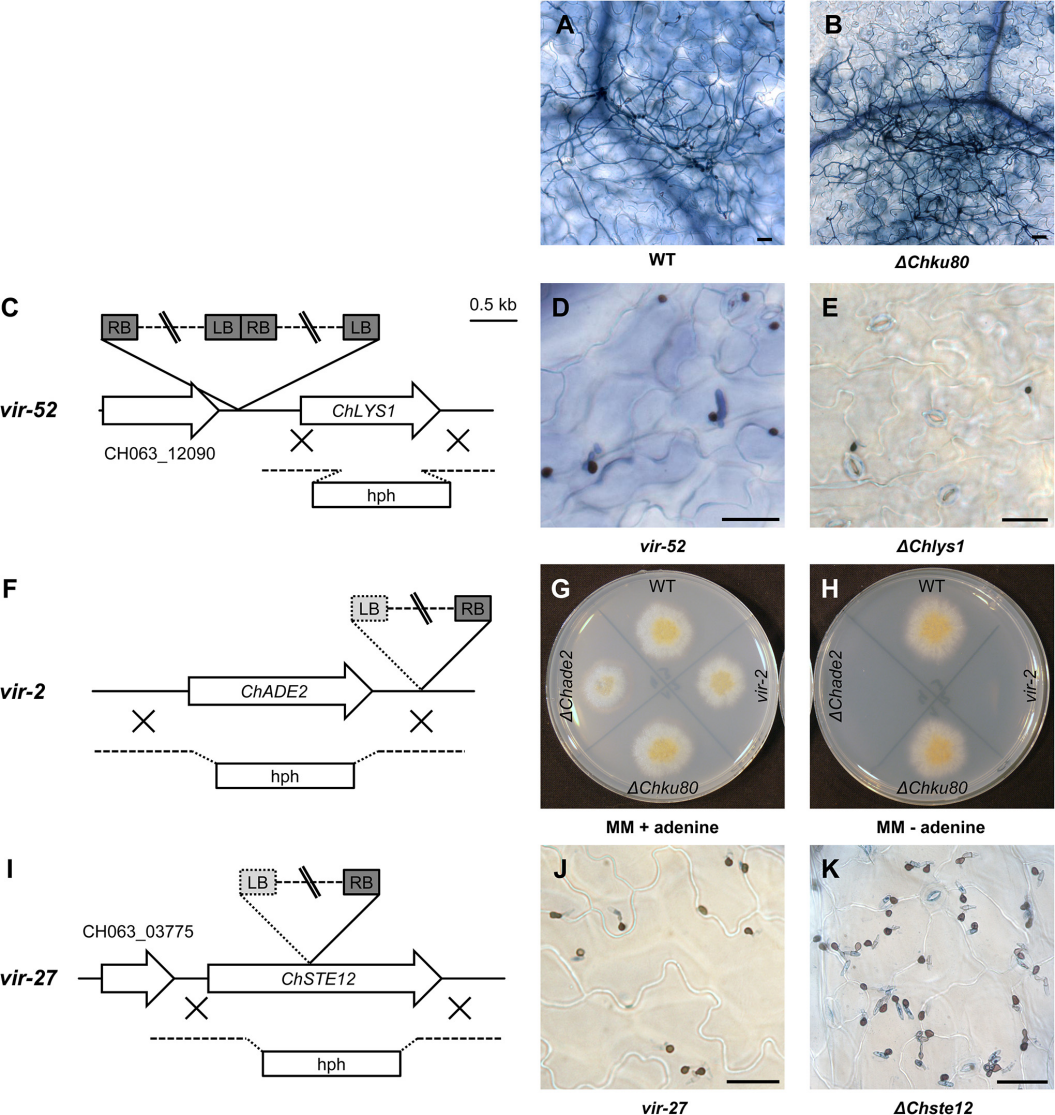
Verification of tagged mutants by targeted knockout. (A, B) Trypan blue stained *A*. *thaliana* leaves four days after spray infection with *C*. *higginsianum* wild type (WT) or *ΔChku80* strain (CY6021). (C, F, I) Schematic representation of T-DNA insertions in genomic loci of three pathogenicity mutants (*vir-52*, *vir-2*, *vir-27*). The respective homology regions used for targeted gene knockout by homologous recombination in the *ΔChku80* strain are illustrated as dotted lines. The T-DNA border sequences identified by Genome Walker PCR are illustrated in dark gray. (D, E) Light microscopy of trypan blue stained *A*. *thaliana* Col-0 leaves four days after spray infection with *vir-52* and *ΔChlys1*. (G, H) Czapek Dox minimal medium without adenine or supplemented with 55 μg/ml adenine four days after inoculation with wild type (WT), *ΔChku80*, *vir-2* or *ΔChade2* strains. (J, K) Light microscopy images of trypan blue stained leaves four days after spray infection with *vir-27* or *ΔChste12*. Scale bar = 25 μm.

ATMT has repeatedly been used for the generation of mutants in filamentous fungi, including *Colletotrichum* species [5–8]. To validate that the integrating T-DNA is responsible for the observed phenotypes, complementation experiments or the generation of targeted knockout mutants are important. This was particularly relevant as a significant number of the isolated *vir* mutants had more than one insertion. Gene disruption in filamentous fungi is generally inefficient compared to *S*. *cerevisiae* [1]. After it was shown that inactivation of components from the non-homologous end-joining pathway (NHEJ) dramatically increased the rate of homologous insertions over random insertions in *Neurospora crassa* [56], this strategy proved successful for gene replacement in several filamentous fungi including *Magnaporthe grisea* [57], *Botrytis cinerea* [58], *Aspergillus sojae* and *A*. *oryzae* [59]. Disruption of *ChKU70* [60] led to an improved frequency of homologous recombination in *C*. *higginsianum* without effects on pathogenicity and growth. We inactivated ChKu80 by replacing most of the coding region by the nourseothricin resistance (*nat*) under the control of the *C*. *higginsianum TRPC* promoter and terminator (S4A Fig). The *ChKU80* locus (CH063_02085) was identified by BLAST analysis with the *Magnaporthe grisea* MgKu80 sequence (XP_365937.1). The predicted proteins exhibit 55% sequence identity. For the deletion construct the *nat* gene was flanked by 800 bp of *ChKU80* upstream and downstream sequences, respectively. The T-DNA contained an additional hygromycin resistance cassette next to the *ChKU80* deletion cassette (S4A Fig) allowing to discriminate homologous recombination from illegitimate insertions. *C*. *higginsianum* wild type strain CY5535 was transformed with the *ChKU80* deletion construct. Out of 400 nourseothricin resistant transformants 60 were sensitive towards hygromycin (15%). After pre-selection by PCR analysis (data not shown), two of these mutants were subjected to Southern blot analysis using a *nat* probe (S4B–C Fig). Both analyzed strains showed the bands expected for successful gene replacement. The phenotype of these two independent *ΔChku80* strains (CY6021, CY6022) were analyzed and showed similar growth, *in vitro* appressoria formation, pathogenicity and induction of host defense reactions as the congenic wild type strain (Fig 4A and 4B; S4D–S4M Fig).

### Verification of tagged mutants by targeted gene knockout

In an ongoing effort to investigate whether the T-DNA insertions identified in Table 2 are responsible for the respective *vir* phenotypes, we tested three mutants by targeted knockout of selected genes. This also allowed us to test the efficiency of homologous recombination in the *ΔChku80* background. Plasmids having all or most of the respective coding regions replaced with a hygromycin resistance gene (Fig 4C, 4F and 4I) were constructed for genes potentially affected in *vir-2*, *vir-52* and *vir-27* (S1 Text). When the resulting plasmids were used for transformation of *ΔChku80* strain CY6021, we found 60 to 90% of all transformants had undergone homologous recombination when analyzed by diagnostic PCR reactions (data not shown, S1 Text). The rate of successful gene targeting was similar for the different constructs and comparable to rates found for *ΔKU70* deletion strains of *C*. *higginsianum* (70–90% [60]) and related fungi (*A*. *sojae* and *A*. *oryzae* 60–90% [59]; *M*. *grisea* >80% [57]).

***Vir-52*:** Targeted gene knockouts were performed for the putative oxidoreductase (CH063_12090) and the *ChLYS1* gene (CH063_02404). Deletion of CH063_12090 did not result in any observable pathogenicity phenotype (not shown). The available annotation for *ChLYS1* (CH063_02404) is incomplete as it is missing its 5’-end. A revised gene model based on the *C*. *graminicola* sequence suggested that the T-DNA insertion is located in the potential promoter region. Replacing *ChLYS1* with the hygromycin resistance had strong phenotypical effects. While *vir-52* was still able to form appressoria and some primary hyphae (Fig 4E), *ΔChlys1* formed only few appressoria *in planta*, which were incapable to penetrate (Fig 4F). Furthermore, this deletion mutant was auxotrophic for lysine (S7 Fig) in contrast to *vir-52*. The more severe phenotype of *ΔChlys*1 could be due to a weak allele of *ChLYS1 in vir-52* instead of a null allele, since the T-DNA insertion is located in the upstream region of *ChLYS1*.

***Vir-2*:** *ΔChade2* deletion mutants (Fig 4F) were generated by homologous recombination. The knockout mutants displayed adenine auxotrophy (Fig 4G and 4H) and like *vir-2* showed reduced *in vitro* appressoria formation (<10%). *ΔChade2* formed black colonies on oatmeal agar medium (S7A Fig). *Vir-2* is less pigmented than *ΔChade2*, which may indicate that ChAde2 has residual activity in *vir-2*. The phenotype of *ΔChade2* seems to be more pronounced than the reported phenotype of *ΔMoade1* mutants from *Magnaporthe oryzae*, which were still able to form appressoria and penetrate but failed to establish biotrophic growth [61]. Mutants of *Fusarium oxysporum* with defects in purine biosynthesis also showed only reduced pathogenicity [62].

***Vir-27*:** *Vir-27* has two independent T-DNA insertions, one inside the ORF CH063_13013 and one in the ORF of *ChSTE12*. Since *vir-27* exhibited a very similar phenotype to *Δcst1* mutants of *C*. *orbiculare* [47], only *ChSTE12* was subjected to targeted gene replacement. The resulting *ΔChste12* strain, like *vir-27*, did not produce primary or secondary hyphae *in planta* (Fig 4K and 4L) strongly suggesting that the pathogenicity phenotype of *vir-27* is caused by loss of ChSte12 function.

***Vir-51*:** Southern blot analysis (data not shown) showed only one band hybridizing to T-DNA, suggesting that the mutant carries a single T-DNA insertion which was identified downstream of CH063_06511 (Table 2). However, targeted deletion of CH063_06511 and the region downstream of CH063_06511 did not reproduce the phenotype of *vir-51* (S8B Fig). This demonstrated that the phenotype of this mutant is not due to the identified T-DNA insertion suggesting that not all mutations generated in this mutant screen are tagged by T-DNA insertions.

### Inactivation of *ChPMA2* results in loss of pathogenicity

**Fig 5:**
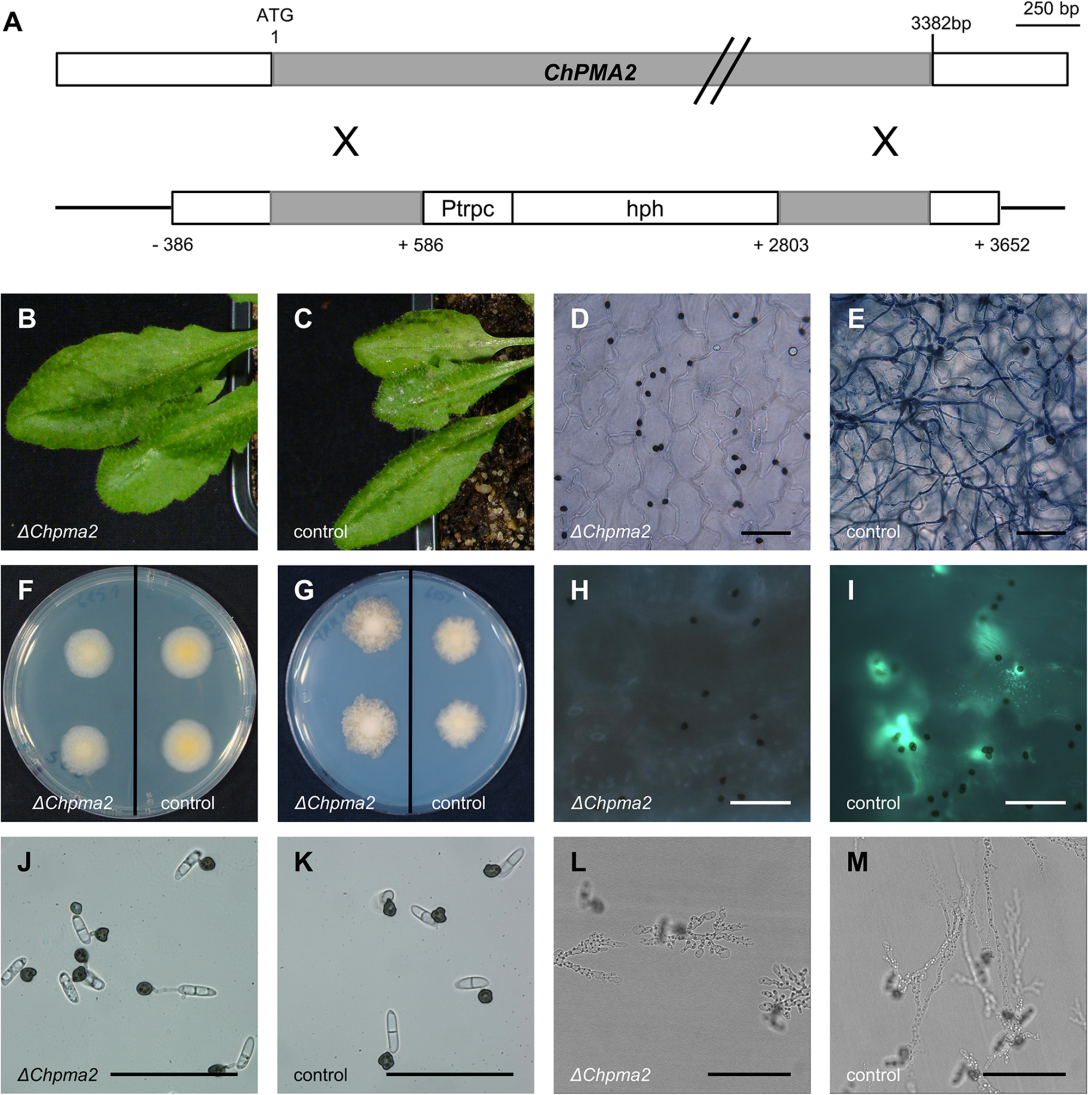
Construction and phenotype of *ΔChpma2* mutants. (A) Schematic overview of the *ChPMA2* locus and the knockout plasmid pCK3349 used for transformation of *C*. *higginsianum* (B, C) Macroscopic symptoms of *A*. *thaliana* leaves four days after spray infection with *C*. *higginsianum ΔChpma2* mutants (B) and the parental *ΔChku80* strain (C, control). (D, E) Trypan blue stained leaves four days after infection with *ΔChpma*2 (D) and the parental *ΔChku80* strain (E). (F, G) Growth of *ΔChpma2* strains compared to the parental strain on PDA medium after 3 days (F) and on minimal medium after five days (G). (H, I) Aniline blue staining of *A*. *thaliana* leaves three days after infection with *ΔChpma*2 and the parental strain (control). (J, K) *In vitro* appressoria of *ΔChpma*2 and the parental strain. (L, M) Penetration of dialysis tubes by the *ΔChpma*2 mutant (L) and the parental strain (M). Scale bar = 50 μm.

The *vir* mutants carrying different insertions in and upstream of *ChPMA2* (see above) showed slightly different phenotypes. Therefore, we generated a knockout allele of *ChPMA2* where most of the coding region of the predicted protein was deleted (i.e. amino acids 158–829). The *ChPMA2* knockout plasmid (pCK3349; S1 Text) was used for ATMT of *ΔChku80 C*. *higginsianum*. The obtained transformants were subjected to diagnostic PCR analysis to verify the gene knockout (S5 Fig). 14 out of 17 transformants showed the product expected for integration at the *ChPMA2* locus, further demonstrating the efficiency of homologous recombination in the *ΔChku80* background. For three transformants the successful recombination and the absence of ectopic integrations was further verified by southern blot analyses (S5 Fig). These three *ΔChpma2* mutants were used for spray inoculation of *A*. *thaliana* plants and showed a phenotype indistinguishable from the two T-DNA insertion mutants *vir-22* and *vir-24* (Fig 5B–5M, S3B Fig) with single insertions in the ORF of *ChPMA2*. Even six days after infection, only appressoria but no primary hyphae were visible in trypan blue stained leaves. This showed that the observed phenotype of *ChPMA2* T-DNA *vir* mutants is caused by the inactivation of *ChPMA2*. This further indicated that *vir-22* and *vir-24* behave like null alleles of *ChPMA2*, while *vir-97* and *vir-102* with insertions in the potential promoter region may encode weak alleles.

### ChPma2 is required for host penetration

**Fig 6:**
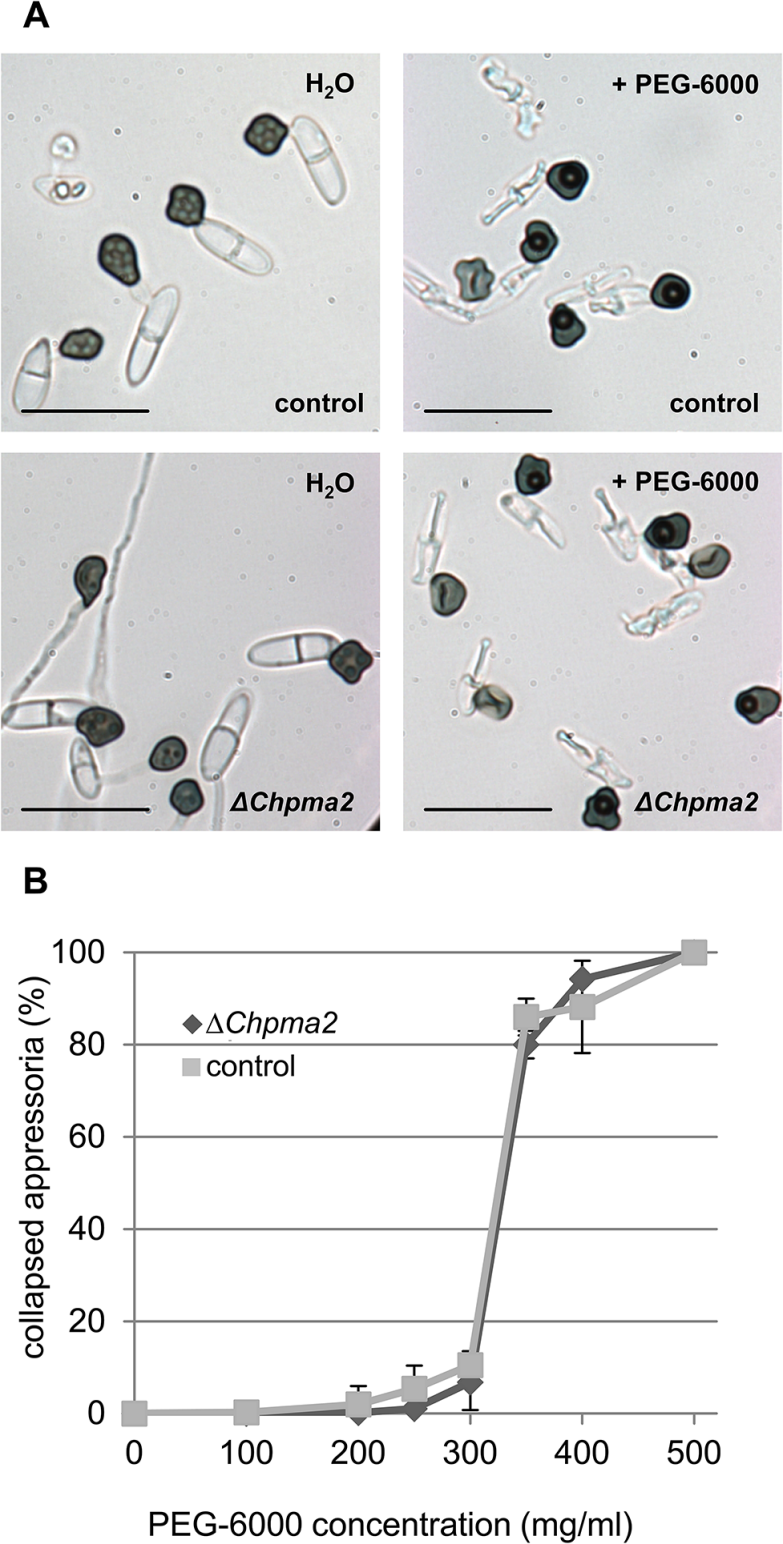
Appressorial turgor pressure in *ΔChpma2* mutants. Appressoria were formed on coated petri dishes. After 24 h, the water was replaced with PEG-6000 solutions ranging from 100 mg/ml to 500 mg/ml. The percentage of collapsed appressoria was determined after 10 minutes of incubation in PEG by phase-contrast microscopy. (A) Typical microscopic images of parental *ΔChku80* (control) and *ΔChpma2* appressoria untreated (H_2_O) or treated with 400 mg/ml PEG-6000 (+ PEG-6000). (B) Percentage of collapsed appressoria of *ΔChpma2* and the parental strain at the corresponding PEG-6000 concentration. Data corresponds to three independent experiments with at least 100 cells counted for each concentration. Scale bar = 15 μm.

To exclude the possibility that the pathogenicity phenotype of *ΔChpma2* mutants was caused by general growth defects, mutants were grown saprophytically on PDA full medium and on minimal media. Both radial agar colonization and colony morphology were similar to the parental *ΔChku80* strain (Fig 5F and 5G). This indicates that *ChPMA2* makes no contribution to vegetative fitness. In order to analyze plant defense reactions, leaves infected with *ΔChpma2* mutants were assayed for callose deposition and ROS production. In contrast to leaves infected with *ΔChku80* and wild type *C*. *higginsianum*, which showed 11% ROS production and 7% callose deposition, *ΔChpma2* knockout mutants did not induce signs of plant defense (Fig 5H and 5I), although the mutants formed fully melanized appressoria on *A*. *thaliana* leaves at 12 h after inoculation. *ΔChpma2* mutants also failed to penetrate epidermis cells and may therefore not be recognized effectively by the host. In order to investigate whether this is a consequence of defective appressoria, their formation was further quantified and analyzed *in vitro*. Like the parental *ΔChku80* strain, *ΔChpma2* mutants showed >90% appressoria formation on petri dishes (Fig 5J and 5K). Furthermore, *ΔChpma2* mutants were able to form appressoria on the surface of dialysis tubes and showed hyphal growth inside the cellulose membrane comparable to the parental strain (Fig 5L and 5M). This illustrates that *ΔChpma2* mutants retain the ability to produce hyphae originating from appressoria on artificial surfaces but fail to do so *in planta*. The appressorial turgor pressure was measured using an incipient cytorrhysis assay with increasing concentrations of the high molecular weight osmolyte PEG-6000 [63, 64]. After plotting the percentage of collapsed appressoria against the PEG-6000 concentration (Fig 6), no differences in cytorrhysis between the *ΔChku80* parental strain and the *ΔChpma2* mutant could be observed. 80% of the appressoria of both strains collapsed at a PEG-6000 concentration of 350 mg/ml which corresponds to an internal turgor pressure of about 2.6 MPa [33]. This value is comparable to the reported appressoria turgor pressure of *C*. *graminicola* [65], indicating that *ΔChpma2* mutants should produce a sufficient turgor pressure for penetration. The infection of wounded tissue with *ΔChpma2* mutants resulted in the production of some vegetative mycelium on the plant surface but no intracellular hyphae. No symptoms could be observed after 7 days (S8 Fig).

### *C*. *higginsianum* encodes two *PMA* paralogs that are differentially expressed

The genome of *Colletotrichum higginsianum* contains a gene very similar to *ChPMA2*, which we called *ChPMA1*. These two proteins share 46% identical residues. Interestingly, the ChPma1 protein is more similar to the *S*. *cerevisiae* plasma membrane ATPases Pma1 (72% identity) and Pma2 (71% identity) than it is to ChPma2, suggesting that ChPma1 and ChPma2 are not functionally identical. Of the two plasma membrane H^+^-transporting ATPases of yeast, ScPma1 is essential [66] while ScPma2 (which is 89% identical to ScPma1) can be inactivated without significantly reducing fitness [67]. A targeted gene knockout of *ChPMA1* was not successful. The few transformants we obtained carried an ectopic integration of the *ChPMA1*-deletion cassette (data not shown), indicating that *ChPMA1* may be an essential gene and may encode the major H^+^-transporting ATPase. In contrast, ChPma2 is not required for vegetative growth but possess specific functions related to pathogenicity.

**Fig 7:**
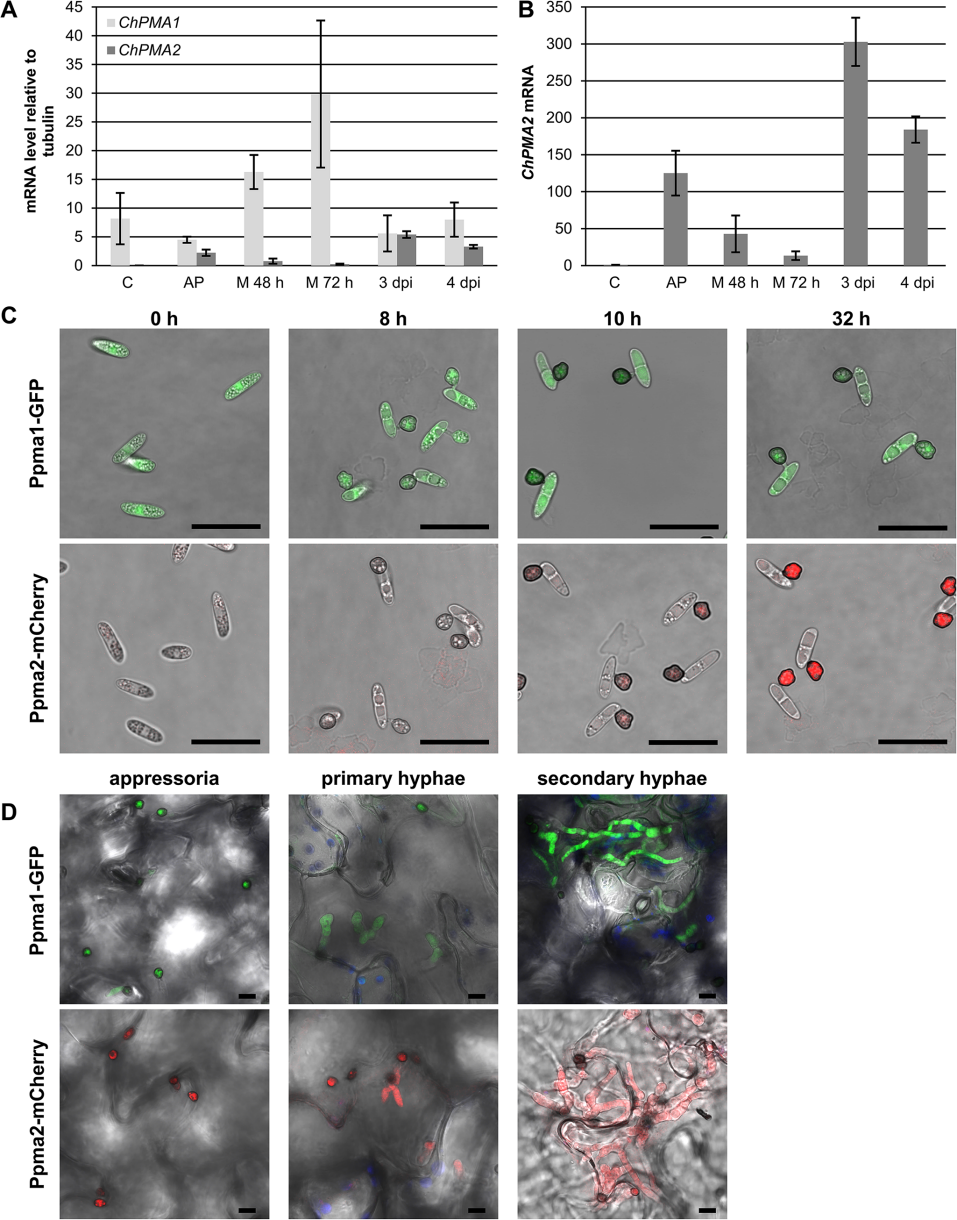
Expression of *ChPMA1* and *ChPMA2*. (A) Quantitative RT-PCR analysis of transcript abundance for *ChPMA1* and *ChPMA2* in conidia, *in vitro* appressoria (AP), mycelium grown 48 h or 72 h in liquid modified Mathur’s medium (M) and during infection of *A*. *thaliana* after three or four days (3 dpi, 4 dpi). Data was normalized against alpha-tubulin (CH063_01222) expression and plotted relative to alpha-tubulin (average from three biological replicates). (B) Normalized *ChPMA2* expression plotted relative to *ChPMA2* level in conidia. (C, D) Confocal images of *C*. *higginsianum* transformed with the promoter fusions Ppma2-mCherry (pCK3880) or Ppma1-GFP (pCK3973) after 0 to 32 h of incubation on coated ibidi μ-dishes (C) or after one and three days of spray infection of *A*. *thaliana* (D). The images are depicted as overlays of bright-field channel with mCherry or GFP-channel (C) or with an additional chloroplast autofluorescence channel in blue (D). Scale bar = 10 μm.

As described above, the phenotype of *ΔChpma2* knockout mutants indicates a specific role for *ChPMA2* in early stages of infection, which may also be reflected by its expression profile. mRNA levels of *ChPMA1* and *ChPMA2* were quantified in conidia, during *in vitro* appressoria formation, in vegetative mycelium grown in modified Mathur’s medium and *in planta* using qPCR analysis (Fig 7A). *ChPMA1* was highly expressed in all samples including conidia. Expression relative to the reference transcript α-tubulin (CH063_01222) was highest when vegetative mycelium was growing in glucose containing media. In contrast, *ChPMA2* mRNA amount was nearly not detectable in conidia and only weakly expressed in vegetative mycelium. Furthermore, *ChPMA2* mRNA was highly expressed in appressoria and *in planta* (Fig 7A). In appressoria, *ChPMA2* levels reached about half of the transcript level of *ChPMA1*. During infection, both *ChPMA1* and *ChPMA2* are expressed at similar levels. ChPma1 may therefore provide the vast majority of the required proton transport in mycelia and conidia, while ChPma2 may become important in appressoria and during infection, which would be consistent with the penetration phenotype of the *ΔChpma2* mutant. The timing of *ChPMA2* expression was further monitored using expression of mCherry under control of the *ChPMA2* promoter (Fig 7C). *ChPMA2* promoter activity was not detectable until *in vitro* appressoria were fully melanized ten hours after induction on petri dishes. In contrast, expression of GFP driven by the *ChPMA1* promoter was visible in conidia and in newly formed, unmelanized appressoria. *In planta* expression of both reporter genes was detected in appressoria, biotrophic and secondary hyphae (Fig 7 D). Expression of *ChPMA2 in planta* was also reported in a genome wide expression study [17].

### *PMA2* is conserved in phytopathogenic fungi

**Fig 8:**
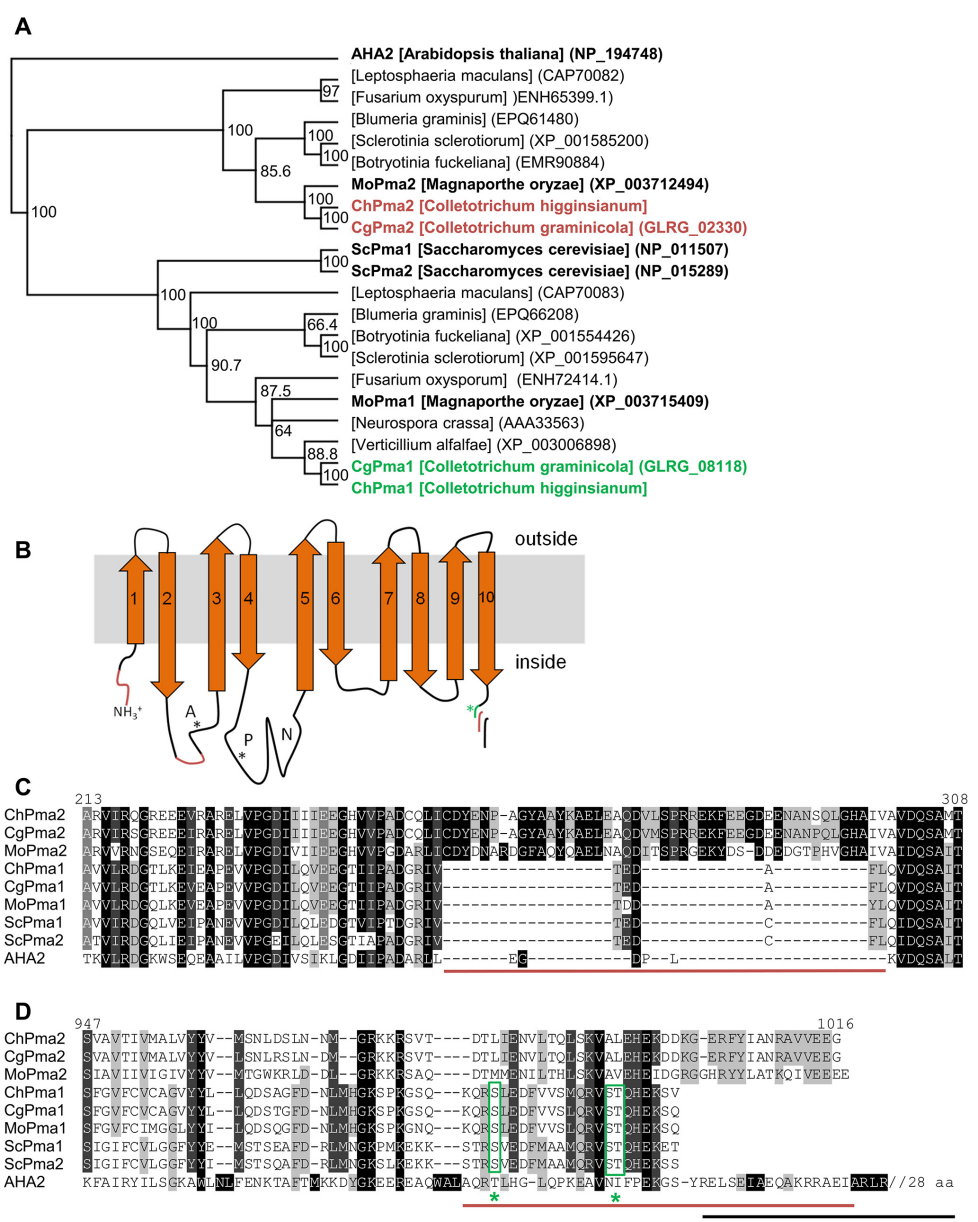
Fungal H^+^-transporting P-type ATPases. (A) Phylogenetic analysis of plasma membrane H^**+**^-ATPases. Sequences were aligned with ClustalW and the tree was generated by Geneious treebuilding (Jukes-Cantor; Neighbor-joining) with AHA2 from *A*. *thaliana* as outgroup. Bootstrap values (1000 replicates) are indicated as percentage at the right side of the nodes. (B) Schematic illustration of the domain structure of H^**+**^-P-type ATPases as found in AHA2 (A: actuator domain, P: phosphorylation domain, N: nucleotide binding domain). Regions specific for ChPma2-like proteins are depicted in red. The green asterisk marks regulatory phosphorylation sites in yeast Pma1. The variable C-terminus is illustrated in red for ChPma2-like proteins, in green for Pma1-like proteins and in black for the autoinhibitory region of Arabidopsis AHA2. (C/D) Amino acid sequence alignment of Pma1 and Pma2 proteins from *Colletotrichum higginsianum* (Ch), *Colletotrichum graminicola* (Cg), *Magnaporthe oryzae* (Mo), *Saccharomyces cerevisiae* (Sc) and *Arabidopsis thaliana* (AHA2). (C) Cytoplasmic region between transmembrane domains two and three. (D) C-terminal region. Potential regulatory serine and threonine residues are marked with green asterisks.

BLAST and phylogenetic analysis were conducted to compare potential H^+^-transporting ATPases between phytopathogenic and nonpathogenic fungi. Using ChPma1, ChPma2 and yeast Pma1 as queries the same set of highly similar sequences (average 50% identity) were obtained, all having more than 28% identities in any crosswise comparison (Fig 8A, S6 Fig). Alignment of sequences to the known structure of the *A*. *thaliana* AHA2 ATPase [68] showed that all sequences contain the conserved domain structure typical for P-Type ATPases such as 10 transmembrane helices as found in Pma-1 of *N*. *crassa* [69] and AHA2 [68]. Furthermore, they clearly belong to the subfamily of H^+^-translocating P-type ATPases from fungi and plants (Fig 8B). ChPma2 shares only little sequence identity with P-type ATPases transporting Ca^2+^ (18% identity to CH063_00398) or Na^+^ (11% identity to CH063_11329). The amino acids reported to form the substrate binding pocket of *N*. *crassa* PMA-1 (Thr^733^, Asp^730^, Glu^805^, Tyr^694^, Arg^695^, Ser^699^ and His^701^) are mostly conserved in all PMA-like ATPases. Interestingly, Ser^699^ is substituted by cysteine and Glu^805^ is substituted by glutamine in ChPma2. ChPma2 is most closely related to ChPma2-like proteins from *C*. *graminicola*, *M*. *oryzae* and *C*. *orbiculare* whereas ChPma1 belongs to a second clade together with the proton pumps from *S*. *cerevisiae*, *N*. *crassa* and *S*. *pombe*.

*S*. *cerevisiae* and *S*. *pombe* have two proton pumps that share high sequence similarity and most likely originated from genome duplications. *Sordaria macrospora*, a non-pathogenic filamentous fungus possesses only one Pma protein with high similarity to yeast Pma1 and Pma2. In contrast, most phytopathogenic ascomycete fungi encode two Pma-like proteins, which can be divided in two distinct clusters by phylogenetic analysis (Fig 8A, S6 Fig). One Pma-like ATPase is most similar to ChPma1 and clusters together with the *S*. *cerevisiae* proton pumps and with *N*. *crassa* Pma-1 (>60% amino acid identity). The second ATPase has features distinct from yeast Pma1 and clusters together with ChPma2. The major sequence differences between Pma1 and Pma2 proteins of phytopathogenic fungi are additional stretches at the N-terminus, at the actuator domain and at the C-terminus of ChPma2 proteins (Fig 8B and 8C). For example, conserved phosphorylation sites at the C-terminus of Pma1 proteins, which regulate activity in response to glucose in yeast [70–72], are missing in ChPma2-like proteins (Fig 8D). The Pma2 cluster can be further divided in two groups according to the infection strategy of the corresponding fungi: On the one hand appressoria-forming pathogens like Colletotrichum species, *Magnaporthe grisea* and *Blumeria graminis* and on the other hand non appressoria-forming phytopathogenic fungi such as *Fusarium oxysporum* and *Leptosphaeria maculans*.

## Discussion

We isolated 75 mutants of *Colletotrichum higginsianum* with defects in pathogenicity which corresponds to approximately 1% of the initially screened transformants. This percentage is comparable to other screens that used ATMT to generate pathogenicity mutants of phytopathogenic fungi. These screens resulted in 4% pathogenicity mutants in *Leptosphaeria maculans* [73], 5% in *Botrytis cinerea* [3], 1% in *Fusarium oxysporum* [74] and from 0.1% to 3% in *Magnaporthe oryzae* [4, 75]. Analogous screens in *C*. *higginsianum* produced similar results with 0.7% [7] and 0.5% [8] virulence mutants. Of the 75 obtained *vir* mutants, we could classify five as mutants with defects in appressoria formation, 19 as penetration mutants and 17 as mutants that do not switch to necrotrophic growth after establishment of biotrophy. In a similar screen of *C*. *higginsianum* that resulted in 40 pathogenicity mutants, Huser et al. [8] identified a large proportion of penetration mutants. Several mutants from previous screens were affected in melanization including *ChMEL1* or induced strong host defense reactions [7, 8]. In contrast, we obtained only two mutants that showed complete or partial loss of melanization (*vir-28* and *vir-11*) and only few that induce more ROS or callose papilla production than the wild type. These differences may reflect the small number of mutants identified so far and different screening conditions. In addition, different fungal isolates were used as parental strains and different *A*. *thaliana* ecotypes for infection. While *Arabidopsis* Col-0 was used in our screen, the *Arabidopsis* ecotype Ler-0 was used by Huser et al. [9]. Ler-0 is described to be more susceptible towards *C*. *higginsianum* than Col-0 and showed stronger induction of host defense reactions [76].

Southern Blot analysis of 70 *vir* mutants showed that 31% harbor single T-DNA insertions. The percentage of single T-DNA insertions was relatively low compared to other ATMT screens [6, 74]. Also, a considerable number of our *vir* mutants contain multiple T-DNAs in tandem configuration. Especially head to tail insertions of two T-DNAs were found. The left to right border junctions of some mutants showed sequence similar to previously described T-circles [39, 77], which may be generated by recombination and ligation of T-DNAs prior to insertion. The prevalence of mutants with more than a single T-DNA insertion may be caused by the artificial nature of the ATMT transformation system e.g. the protocol for acetosyringone treatment. The hypervirulent *Agrobacterium* strain we used for ATMT was not reported to have a preference for multiple insertions in other mutant screens [3, 40]. However, it was described as particularly effective for the transformation of filamentous fungi [78]. This observation is consistent with an increased frequency of T-DNA transfer.

Since *C*. *higginsianum* lacks a sexual stage, multiple insertions cannot be separated by meiotic segregation and consequently complicate the assignment of the identified T-DNA insertion to the observed phenotype. Therefore, we generated *Chku80* deficient strains to allow targeted gene disruptions of potential pathogenicity related genes. As previously reported for NHEJ deficient mutants of filamentous fungi like *M*. *grisea* [57], *Botrytis cinerea* [58], *Aspergillus fumigatus* [79] and *C*. *higginsianum* [60], the efficiency of homologous recombination was greatly increased without influencing the phenotype towards pathogenicity and growth fitness.

18 T-DNA insertion sites of *vir* mutants in our screen were identified using adapter ligation-mediated PCR analysis. Among the candidate genes we observed no overlap with genes identified in other *C*. *higginsianum* screens except for the identification of mutants auxotrophic for arginine [7, 8]. However, we found possible orthologs of previously described virulence-related genes in other *Colletotrichum* species like *STE12* [47] and *KEL2* [44]. The missing overlap of genes from different forward genetic screens indicates that the random insertional mutagenesis approach is far from saturated and that the host ecotype may be important. In addition, it is not known for how many insertion mutants the T-DNA insertion is not responsible for the observed phenotype. For the *vir-51* mutant, we observed that neither the deletion of the T-DNA insertion site nor the deletion of the neighboring gene could reproduce the pathogenicity phenotype of the original mutant *vir-51*. Untagged mutants were reported to be a problem in insertional mutagenesis especially in REMI based mutagenesis but also in ATMT screens [1].

Interestingly, T-DNA insertion sites of five separate *vir* mutants could be assigned to a single locus (*ChPMA2*), which represents a high percentage of the identified insertion sites. In a similar screen of *C*. *higginsianum* [8], 2 out of 12 identified T-DNA insertions were in an importin-β2 homolog. Furthermore, a T-DNA mutant library of *F*. *oxysporum* [74] contained two mutants with insertions upstream of *FOW2* and three mutants with insertions in a class V chitin synthase. Meng *et al*. [40] also reported a non-random distribution of insertion events in *Magnaporthe oryzae* and proposed hot spots for integration. *ChPMA2* could represent such a potential hot spot. In addition, since *vir* mutants with the T-DNA integration in the ORF of *ChPMA2* (*vir-12* and *vir-22*) do not show any growth or conidiation defects but have a strong pathogenicity phenotype, they may have been identified very efficiently. The observed phenotype of mutants containing T-DNA insertions in the *ChPMA2* coding region could be reproduced by targeted gene knockout of *ChPMA2*. *A*. *thaliana* elicits no detectable defense when infected with *ΔChpma2* mutants, i.e. no papillae are formed, no reactive oxygen species are detectable and no induction of camalexin was observed (data not shown). Appressorium formation and generation of high turgor pressure is thought not to be sufficient for eliciting full pre penetration defense responses [80]. Additional functions of appressorial maturation like secretion of enzymes and penetration peg formation are important events leading to full induction of plant responses before the first cell is invaded [12, 80, 81]. The apparent absence of defense reactions suggests that the *ΔChpma2* mutants arrest very early. However, the ability to form appressoria on artificial surfaces and the ability of these appressoria to produce invasive hyphae in cellulose membranes is not impaired. In *Leptosphaeria maculans* a potential *ChPMA2* ortholog was reported as a pathogenicity factor. *LmPMA1* [55] was postulated to be important for the generation of turgor pressure in conidia. This is unlikely to be the main cause for the phenotype of *ΔChpma2* mutants since appressoria of these mutants were able to build up turgor pressure comparable to wild type appressoria. These observations suggest that appressoria of *ΔChpma2* mutants fulfill the physical requirements for penetration but fail to do so *in planta* because other processes requiring a large proton motif force across the membrane are not supported. These may include secretion or proton coupled transport. A significant number of membrane transporters is activated in *C*. *higginsianum* upon contact with the host [17] prior to invasion which may be critically dependent on ChPma2 function.

Two properties of ChPma2 support the role of this protein in penetration. First, its expression is absent in conidia and becomes induced when appressoria differentiate. *ChPMA2* remains expressed during infection but is only weakly expressed in vegetative mycelium. Second, the protein sequence of ChPma2 exhibits small but conserved alterations from the canonical structure of the yeast proton pump ScPma1. This argues that both the timing of *ChPMA2* expression and the function of the expressed protein are important. We suggest that ChPma2 is required to sustain the proton gradient under conditions where ChPma1 may become inactivated i.e. in the absence of nutrients. In yeast, Pma1 activity is regulated by glucose induced phosphorylation of Ser^899^, Ser^911^ and Thr^912^ [70, 71]. ChPma1, which is very similar to its yeast ortholog Pma1, shares these conserved regulatory phosphorylation sites and may therefore be similarly regulated by glucose. During the penetration process, which occurs in the absence of external nutrients, ChPma1 enzyme activity may become down regulated by such a mechanism. The respective regulatory region of Pma1-like enzymes is missing in ChPma2 and related proteins, consistent with the possibility that their activities are differently regulated, e.g. insensitive to nutrient control. The sequences at the C- and N-terminus of ChPma2 may provide other means of regulation not present in ChPma1. In Pma-like proteins from plants the C-and N-terminal sequences fulfill regulatory roles involving auto inhibition [82] and phosphorylation dependent binding of 14-3-3-proteins [83]. The conserved features of ChPma2 like proton pumps, including the presence of a conserved loop in the cytoplasmic insertion between membrane spanning regions 2 and 3 (Fig 8B) have not been noticed before. Whether or not ChPma2 has additional functions during later stages of infection is difficult to analyze because the respective mutants never progress that far. The failure of *ΔChpma2* mutants to infect wounded plants (S8 Fig) is, however, not inconsistent with a function for ChPma2 also in later stages of infection. The presence of multiple plasma membrane H^+^-transporting ATPases that are differentially regulated on transcriptional levels may also represent a common strategy of pathogenic fungi. For example, the transcript of the *Magnaporthe grisea PMA2*-like gene MGG_04994 was nearly not detectable in conidia but was found among the genes upregulated during appressoria formation [84, 85]. In contrast, the expression of the ortholog of *ChPMA1*, MGG_07200, showed no altered expression during germination and appressoria formation [85]. In addition, a *L*. *maculans* virulence mutant screen identified a gene encoding LmPma1 [55], which shares more sequence homology with ChPma2 than ChPma1. Although having a specific function in pathogenicity, the expression pattern and strength of *LmPMA1* is more comparable to that of *ChPMA1*. The arbuscular mycorrhiza fungi *Glomus mosseae* encodes at least two H^+^-transporting ATPases, *GmPMA1* and *GmHA5* [86, 87]. *GmHA5* was shown to be regulated in response to nutrition and infection while *GmPMA1* showed no differential expression [87]. *A*. *niger* and *A*. *fumigatus* possess one Pma1-like protein and two Pma2-like proteins. The presence of proton pumps with special activities and their importance for the plant fungal interface has also recently been recognized in plants where specific PMA enzymes (Os-Ha1 and Mt-HA1) from rice and *Medicago* are induced during mycorrhizal symbiosis and are required to provide enhanced proton motive force for nutrient transport across the periarbuscular membrane [88].

In summary, our genetic screen has identified a collection of 75 pathogenicity mutants and several potential candidate genes whose specific roles can now be further analyzed by targeted manipulation in the *ΔChku80* background. The finding that *ChPMA2* encodes a pathogen specific P-type ATPase required early upon infection not only provides an interesting further possibility to analyze the detailed processes during appressorium formation and host penetration, but may also shed light on the regulation of this novel potential proton pump.

## Supporting Information

S1 Fig Symptom development during *C*. *higginsianum* infection of *A*. *thaliana* Col-0 plants. (A-D) Macroscopic progression of infection after 1 dpi (A), 3 dpi (B), 4 dpi (C) and 6 dpi (D). Light microscopic analysis of trypan blue stained leaves after 1 dpi (E), 3 dpi (F), 4 dpi (G) and 6 dpi (H). Spray infection was performed using 1 x 10^6^ conidia/ml of *C*. *higginsianum* wild type. AP = appressorium, PH = primary hypha, SH = secondary hypha. Scale bars = 25 μm.
(TIF)

S2 Fig Determination of T-DNA copy number in *vir* mutants by Southern blot analysis. (A) Structure of the T-DNA in pPK2 containing the *A*. *nidulans* glyceraldehyde-3-phosphate dehydrogenase promotor (Pgdpa), hygromycin resistance gene (*hph*) and the *A*. *nidulans* anthranilate synthase terminator (TtrpC) between left (LB) and right border (RB) sequences. SalI and BamHI restriction sites and the 974 nt *hph* probe are indicated. (B) Southern blots of 17 representative *C*. *higginsianum vir* mutants generated by transformation with pPK2. Genomic DNA was digested either with BamHI (left blot) or SalI (right blot) and hybridized to a α-^32^P-dCTP labeled *hph* probe.
(TIF)

S3 Fig T-DNA insertion at the *ChPMA2* locus. (A) Schematic overview of the *ChPMA2* gene with T-DNA insertion sites of *vir-102*, *vir-97*, *vir-12*, *vir-22 and vir-24* relative to the first ATG of its ORF (= 1). Mutants marked with an asterisk contain an unknown second T-DNA insertion. (B) Macroscopic symptom development and trypan blue staining of *A*. *thaliana* leaves four days after spray inoculation with *C*. *higginsianum* wild type and *ChPMA2* T-DNA insertion mutants. Scale bar = 50 μm.
(TIF)

S4 Fig Construction and characterization of a *Chku80* deletion strain. (A) Schematic illustration of the homologous recombination between the T-DNA of knockout plasmid pCK2831 and the *ChKU80* locus. (B) Predicted restriction map of the *ΔChku80* allele after homologous recombination. Numbers represent the distance in bp between restriction sites. LB (left border), RB (right border), nat (nourseothricin resistance gene), *hph* (hygromycin phosphotransferase). (C) Southern blot analysis of PvuII (left) or XhoI (right) digested genomic DNA from *C*. *higginsianum* wild type strain CY5535 (lane 2), *ΔChku80-1* (CY6021) (lane 3), *ΔChku80-2* (CY6022) (lane 4) and linearized pCK2831 (lane1) hybridized to a α-^32^P-dCTP-labeled *nat* probe. *M* (size marker) (D, E) Development of macroscopic symptoms six days after spray infection of leaves with *C*. *higginsianum* wild type strain CY5535 (D) and *ΔChku80* (CY6021) (E). (F-I) Induction of host defense responses after 3 days of spray infection of *A*. *thaliana* with *ΔChku80* and wild type. Callose deposition was stained with aniline blue (F, G) and diaminobenzidine (DAB) was used for staining of reactive oxygen species (H, I). AP = appressorium, Pa = callose papilla. (J, K) Appressoria formation of *ΔChku80* and wild type on coated petri dishes after 14 h at 25°C. (L, M) Radial growth of *ΔChku80* on minimal medium after 5 days (L) and PDA plates after 3 days (M) compared to wild type. Scale bar = 50 μm.
(TIF)

S5 Fig Confirmation of the *ChPMA2* knockout. (A) Schematic illustration of the *ChPMA2* locus before (WT) and after homologous recombination (*ΔChpma2*) with the T-DNA of pDelPMA2 (pCK3349). Probes used for Southern Blot analysis in B and C are depicted as a black bar (probe 1) or black, striped bar (probe 2). Probe 2 hybridizes with the hygR cassette and the homology regions for recombination. Primers used for PCR analysis in D and F are indicated. Primers c and d only produce a PCR product after integration of the deletion cassette at the *ChPMA2* locus. Genomic sequences 3´ of primer site d are unknown. (B/C) Southern blot analysis of genomic DNA from *ΔChku80* (WT for *PMA2* locus) and from three *ΔChpma2* mutants CY6151 (1), CY6152 (9) and CY6153 (17) digested with EcoRV (panel B) or XbaI plus EcoRV (panel C). (B) Hybridization to a ^32^P labeled EcoRV restriction fragment of *ChPMA2* (probe 1). The expected *ChPMA2* band is 1500 bp. The weak band of about 2400 bp is caused by cross-hybridization and is also present in the wild type. (C) Hybridization to a ^32^P labeled EcoRV fragment of pDelPMA2 (probe 2) containing most of the T-DNA. Expected sizes of hybridizing bands are 1100 bp and 1900 bp for the WT and 900 bp and 3200 bp for the *ΔChpma2* allele. The 3200 bp band can only be generated after homologous recombination, whereas ectopic insertions would generate additional bands of different sizes. (D/E) DNA from 17 strains of *ΔChku80* (CY6021) transformed with plasmid pDelPMA2 was isolated and used for diagnostic PCR. The absence of an internal *ChPMA2* fragment was tested in (D) and the successful integration of the hygromycin resistance cassette into the *ChPMA2* locus was tested in (E). M: 1 kb DNA Ladder, 1–17: genomic DNA of 17 transformants as template (numbering corresponds to panels B and D),-: no template control.
(TIF)

S6 Fig Phylogenetic tree of fungal H^+^-transporting P-type ATPases. Phylogenetic analysis of fungal plasma membrane H^+^-ATPases. Sequences were aligned with ClustalW and the tree was generated by Geneious treebuilding (Jukes-Cantor; Neighbor-joining) with Ca^2+^ ATPase 4 from *A*. *thaliana* (NP_181687) as outgroup. Bootstrap values (1000 replicates) are indicated as percentage at the right side of the nodes.
(TIF)

S7 Fig Phenotypes of auxotrophic mutants. (A-C) Growth of *C*. *higginsianum ΔChade2* (A), wildtype (B) and *vir-2* strains (C) on oatmeal agar plates after seven days. (D-E) Growth of three *C*. *higginsianum ΔChlys1* mutants and the *ΔChku80* parental strain on Czapek Dox minimal medium (D) or on Czapek Dox supplemented with 76 mg/l lysine (E) after six days.
(TIF)

S8 Fig Phenotypes of *C*. *higginsianum* deletion mutants. (A) Schematic representation of the T-DNA insertion site of *vir-51*. The homology regions used for targeted gene knockout by homologous recombination in the *ΔChku80* strain are illustrated as dotted lines. The T-DNA border sequence identified by Genome Walker PCR is illustrated in dark gray. (B) *A*. *thaliana* plants four days after spray infection with *ΔChku80*, *vir-51* and *ΔCH063_06511* strains. (C) Wounded *A*. *thaliana* leaves 3 days after droplet inoculation with *ΔChku80*, *ΔChpma2* or mock control (H_2_O). The area around the wounding site is shown after trypan blue staining. (D) Wounded *A*. *thaliana* leaves 7 days after droplet infection. The left side of each leaf was perforated with a single hole using a small pin, while the right side was left unwounded.
(TIF)

S1 Text Supporting materials and methods. Contains details about plasmid constructions, DNA primers and additional methods.
(DOCX)
