# Supplementary figures and images for: A Genetic Screen for Pathogenicity Genes in the Hemibiotrophic Fungus Colletotrichum higginsianum Identifies the Plasma Membrane Proton Pump Pma2 Required for Host Penetration
Source: PLoS One. 2015 May 19;10(5):e0125960. doi: 10.1371/journal.pone.0125960 (PMC4437780; doi:10.1371/journal.pone.0125960)

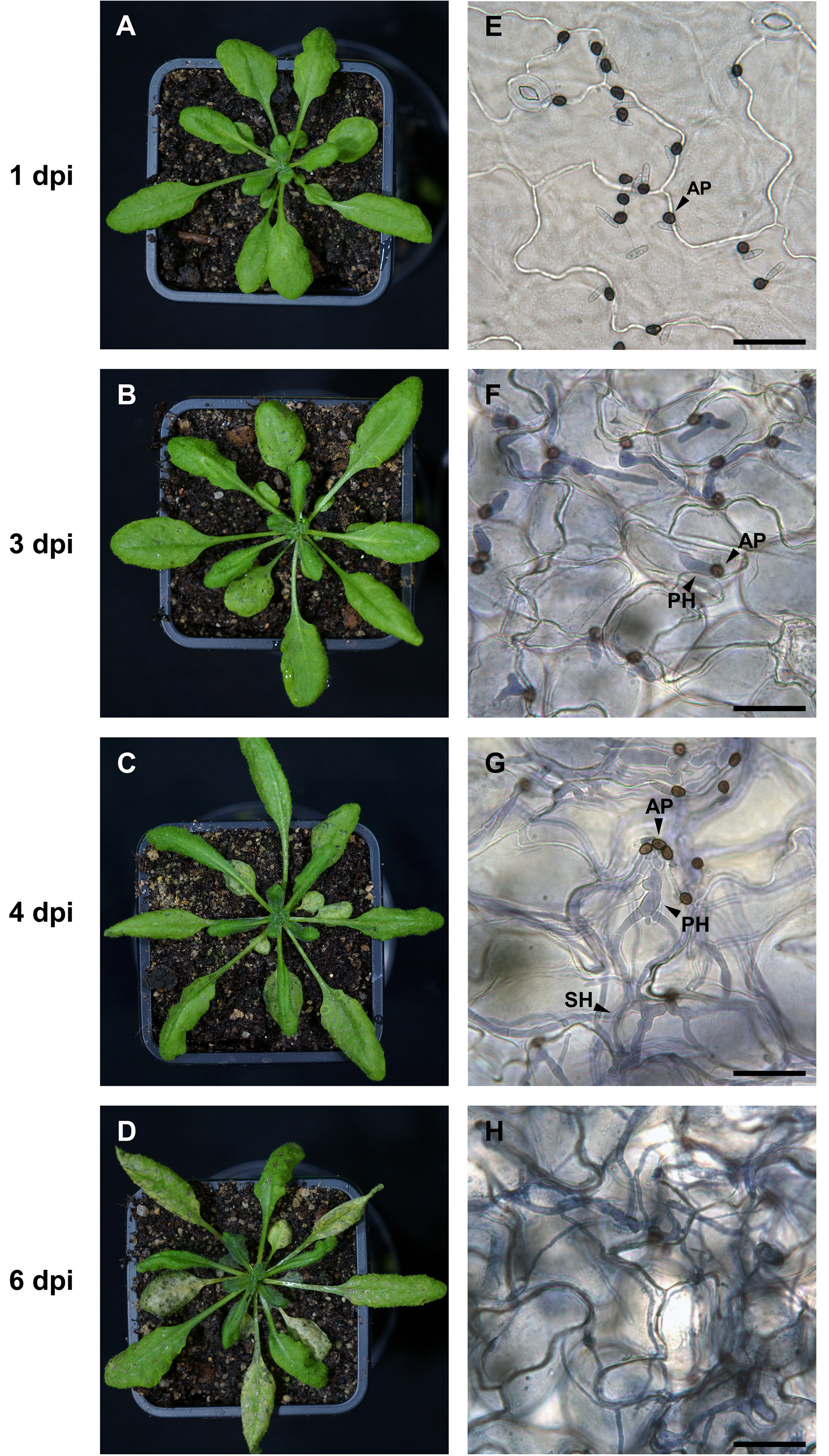

Supplement: S1 Fig — (A-D) Macroscopic progression of infection after 1 dpi (A), 3 dpi (B), 4 dpi (C) and 6 dpi (D). Light microscopic analysis of trypan blue stained leaves after 1 dpi (E), 3 dpi (F), 4 dpi (G) and 6 dpi (H). Spray infection was performed using 1 x 10^6^ conidia/ml of C. higginsianum wild type. AP = appressorium, PH = primary hypha, SH = secondary hypha. Scale bars = 25 μm. (TIF) [file pone.0125960.s001.tif]

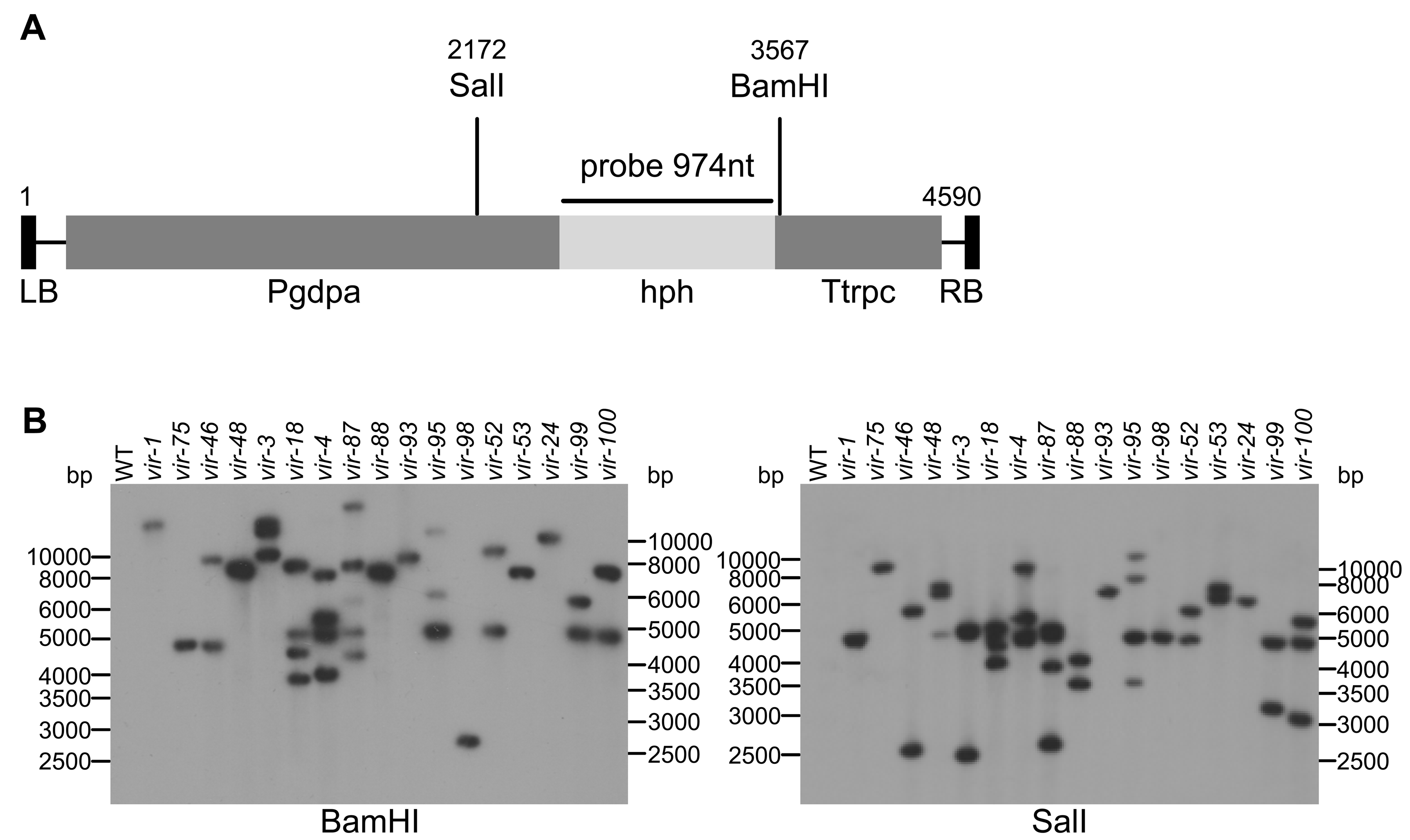

Supplement: S2 Fig — (A) Structure of the T-DNA in pPK2 containing the A. nidulans glyceraldehyde-3-phosphate dehydrogenase promotor (Pgdpa), hygromycin resistance gene (hph) and the A. nidulans anthranilate synthase terminator (TtrpC) between left (LB) and right border (RB) sequences. SalI and BamHI restriction sites and the 974 nt hph probe are indicated. (B) Southern blots of 17 representative C. higginsianum vir mutants generated by transformation with pPK2. Genomic DNA was digested either with BamHI (left blot) or SalI (right blot) and hybridized to a α-32P-dCTP labeled hph probe. (TIF) [file pone.0125960.s002.tif]

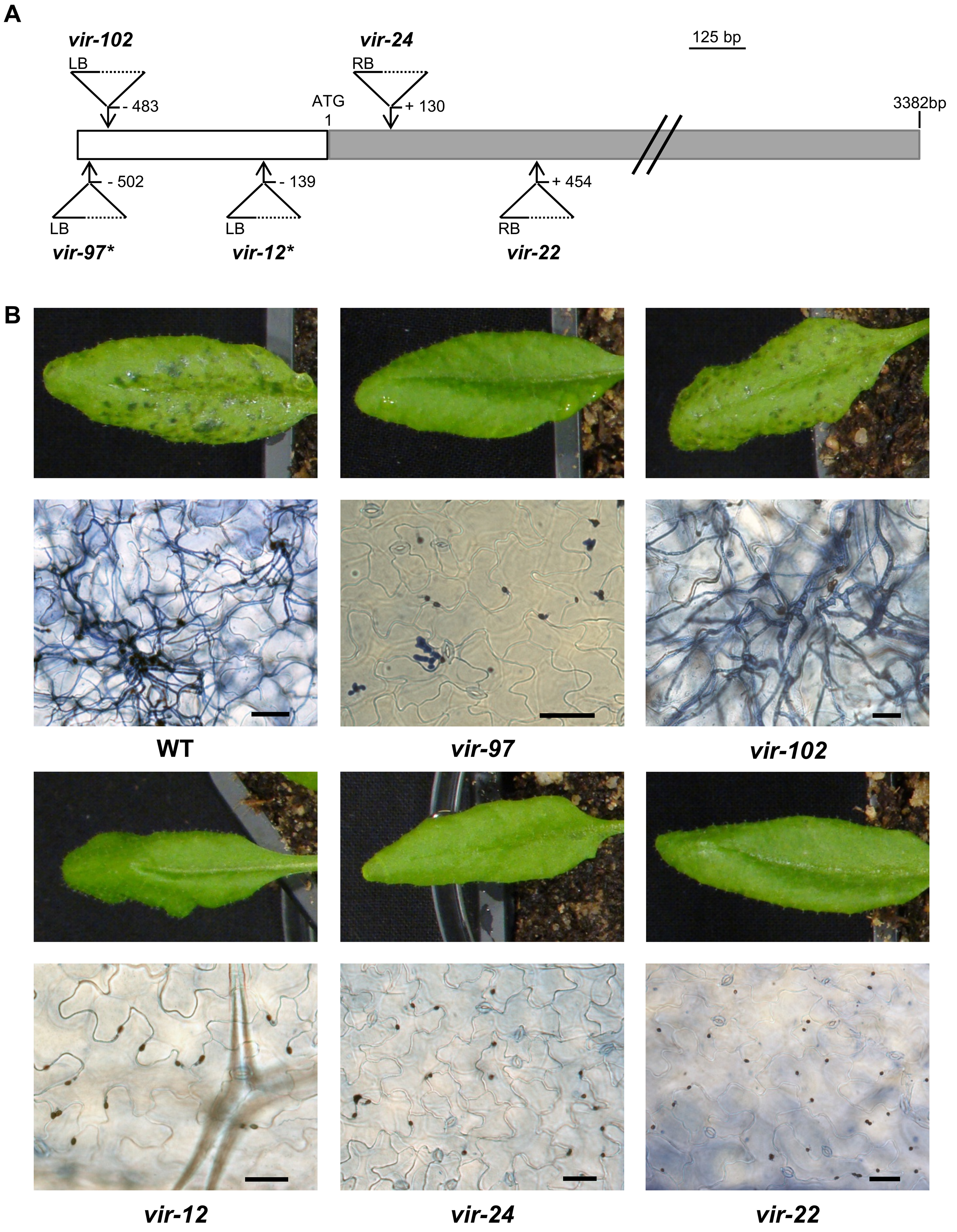

Supplement: S3 Fig — (A) Schematic overview of the ChPMA2 gene with T-DNA insertion sites of vir-102, vir-97, vir-12, vir-22 and vir-24 relative to the first ATG of its ORF (= 1). Mutants marked with an asterisk contain an unknown second T-DNA insertion. (B) Macroscopic symptom development and trypan blue staining of A. thaliana leaves four days after spray inoculation with C. higginsianum wild type and ChPMA2 T-DNA insertion mutants. Scale bar = 50 μm. (TIF) [file pone.0125960.s003.tif]

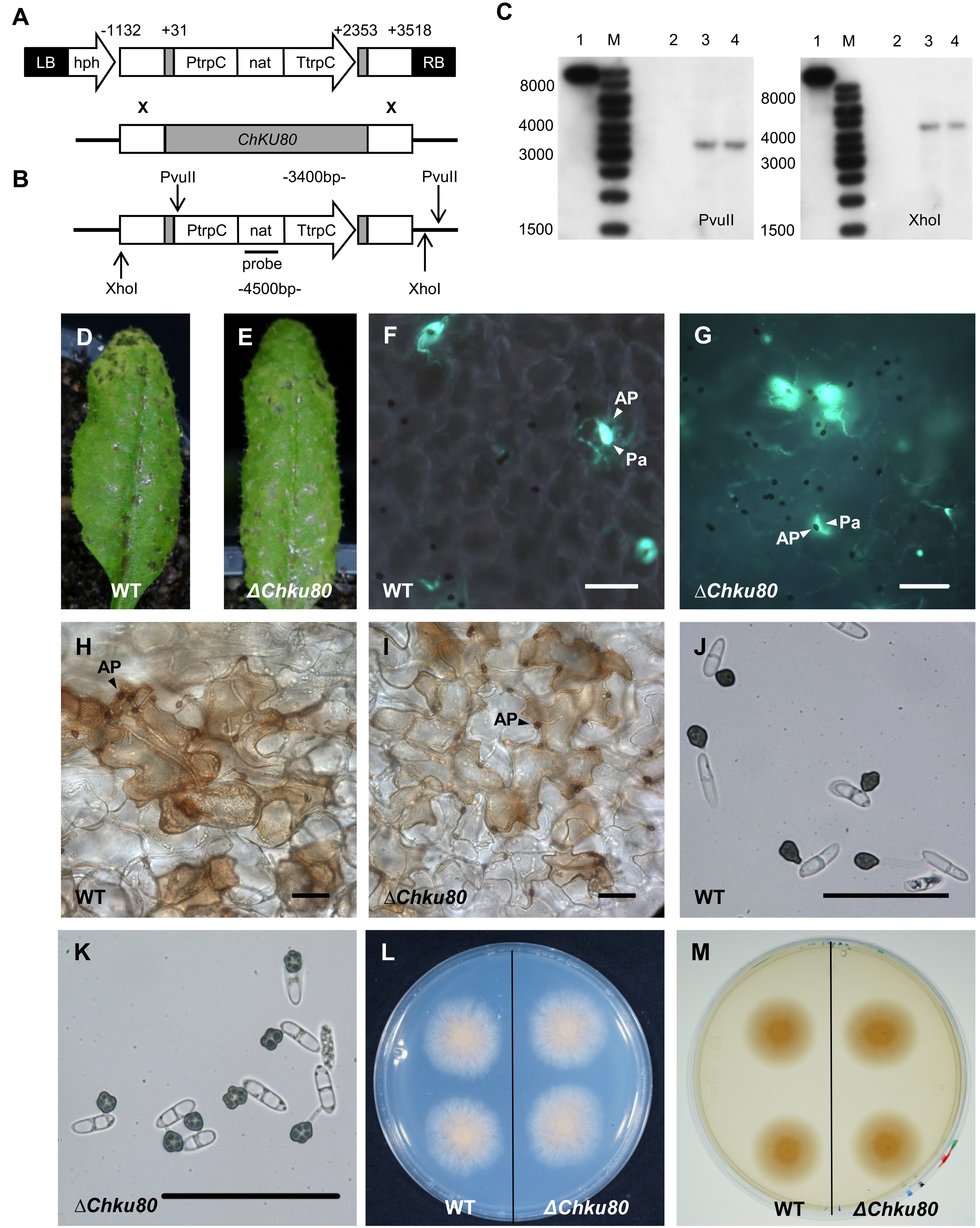

Supplement: S4 Fig — (A) Schematic illustration of the homologous recombination between the T-DNA of knockout plasmid pCK2831 and the ChKU80 locus. (B) Predicted restriction map of the ΔChku80 allele after homologous recombination. Numbers represent the distance in bp between restriction sites. LB (left border), RB (right border), nat (nourseothricin resistance gene), hph (hygromycin phosphotransferase). (C) Southern blot analysis of PvuII (left) or XhoI (right) digested genomic DNA from C. higginsianum wild type strain CY5535 (lane 2), ΔChku80-1 (CY6021) (lane 3), ΔChku80-2 (CY6022) (lane 4) and linearized pCK2831 (lane1) hybridized to a α-32P-dCTP-labeled nat probe. M (size marker) (D, E) Development of macroscopic symptoms six days after spray infection of leaves with C. higginsianum wild type strain CY5535 (D) and ΔChku80 (CY6021) (E). (F-I) Induction of host defense responses after 3 days of spray infection of A. thaliana with ΔChku80 and wild type. Callose deposition was stained with aniline blue (F, G) and diaminobenzidine (DAB) was used for staining of reactive oxygen species (H, I). AP = appressorium, Pa = callose papilla. (J, K) Appressoria formation of ΔChku80 and wild type on coated petri dishes after 14 h at 25°C. (L, M) Radial growth of ΔChku80 on minimal medium after 5 days (L) and PDA plates after 3 days (M) compared to wild type. Scale bar = 50 μm. (TIF) [file pone.0125960.s004.tif]

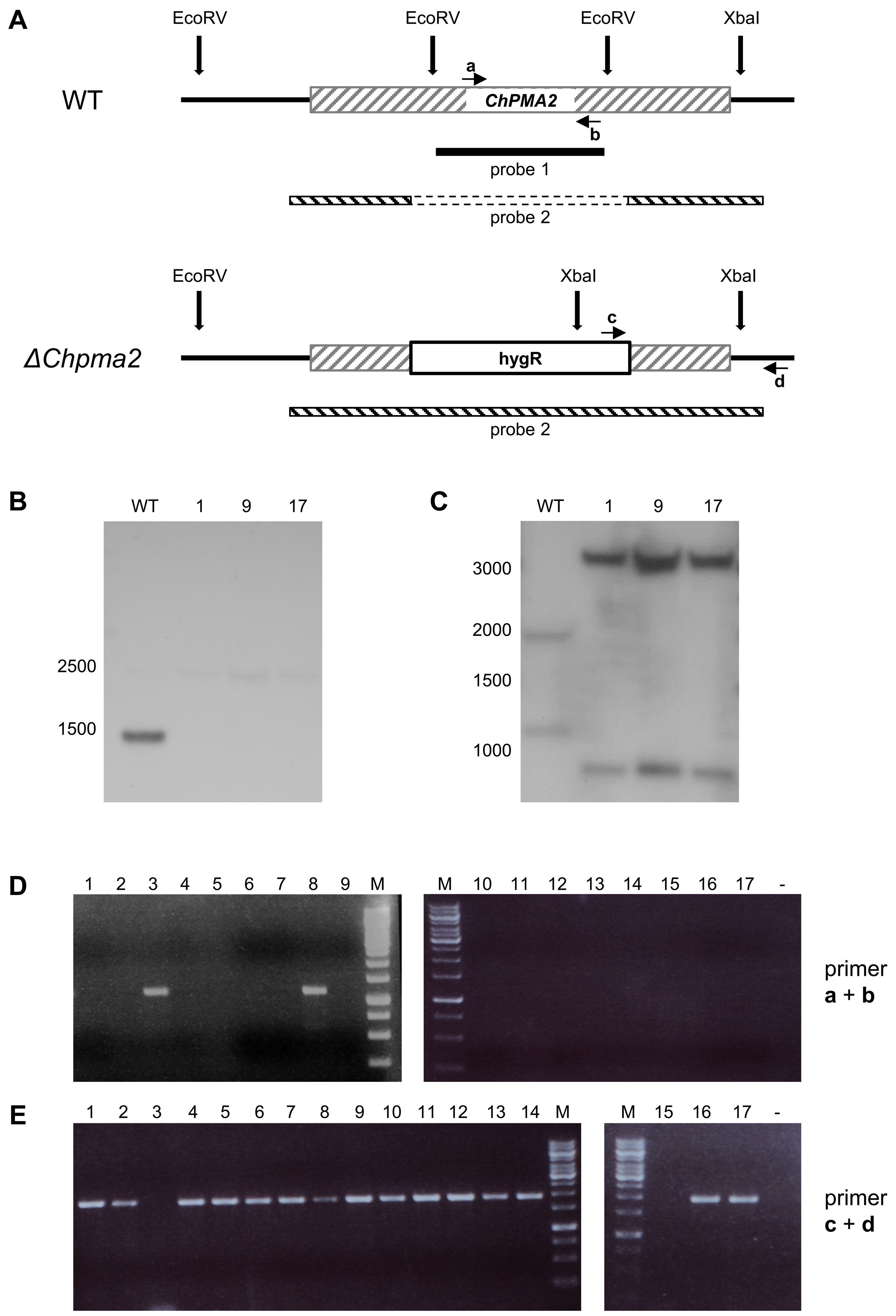

Supplement: S5 Fig — (A) Schematic illustration of the ChPMA2 locus before (WT) and after homologous recombination (ΔChpma2) with the T-DNA of pDelPMA2 (pCK3349). Probes used for Southern Blot analysis in B and C are depicted as a black bar (probe 1) or black, striped bar (probe 2). Probe 2 hybridizes with the hygR cassette and the homology regions for recombination. Primers used for PCR analysis in D and F are indicated. Primers c and d only produce a PCR product after integration of the deletion cassette at the ChPMA2 locus. Genomic sequences 3´ of primer site d are unknown. (B/C) Southern blot analysis of genomic DNA from ΔChku80 (WT for PMA2 locus) and from three ΔChpma2 mutants CY6151 (1), CY6152 (9) and CY6153 (17) digested with EcoRV (panel B) or XbaI plus EcoRV (panel C). (B) Hybridization to a 32P labeled EcoRV restriction fragment of ChPMA2 (probe 1). The expected ChPMA2 band is 1500 bp. The weak band of about 2400 bp is caused by cross-hybridization and is also present in the wild type. (C) Hybridization to a 32P labeled EcoRV fragment of pDelPMA2 (probe 2) containing most of the T-DNA. Expected sizes of hybridizing bands are 1100 bp and 1900 bp for the WT and 900 bp and 3200 bp for the ΔChpma2 allele. The 3200 bp band can only be generated after homologous recombination, whereas ectopic insertions would generate additional bands of different sizes. (D/E) DNA from 17 strains of ΔChku80 (CY6021) transformed with plasmid pDelPMA2 was isolated and used for diagnostic PCR. The absence of an internal ChPMA2 fragment was tested in (D) and the successful integration of the hygromycin resistance cassette into the ChPMA2 locus was tested in (E). M: 1 kb DNA Ladder, 1–17: genomic DNA of 17 transformants as template (numbering corresponds to panels B and D),-: no template control. (TIF) [file pone.0125960.s005.tif]

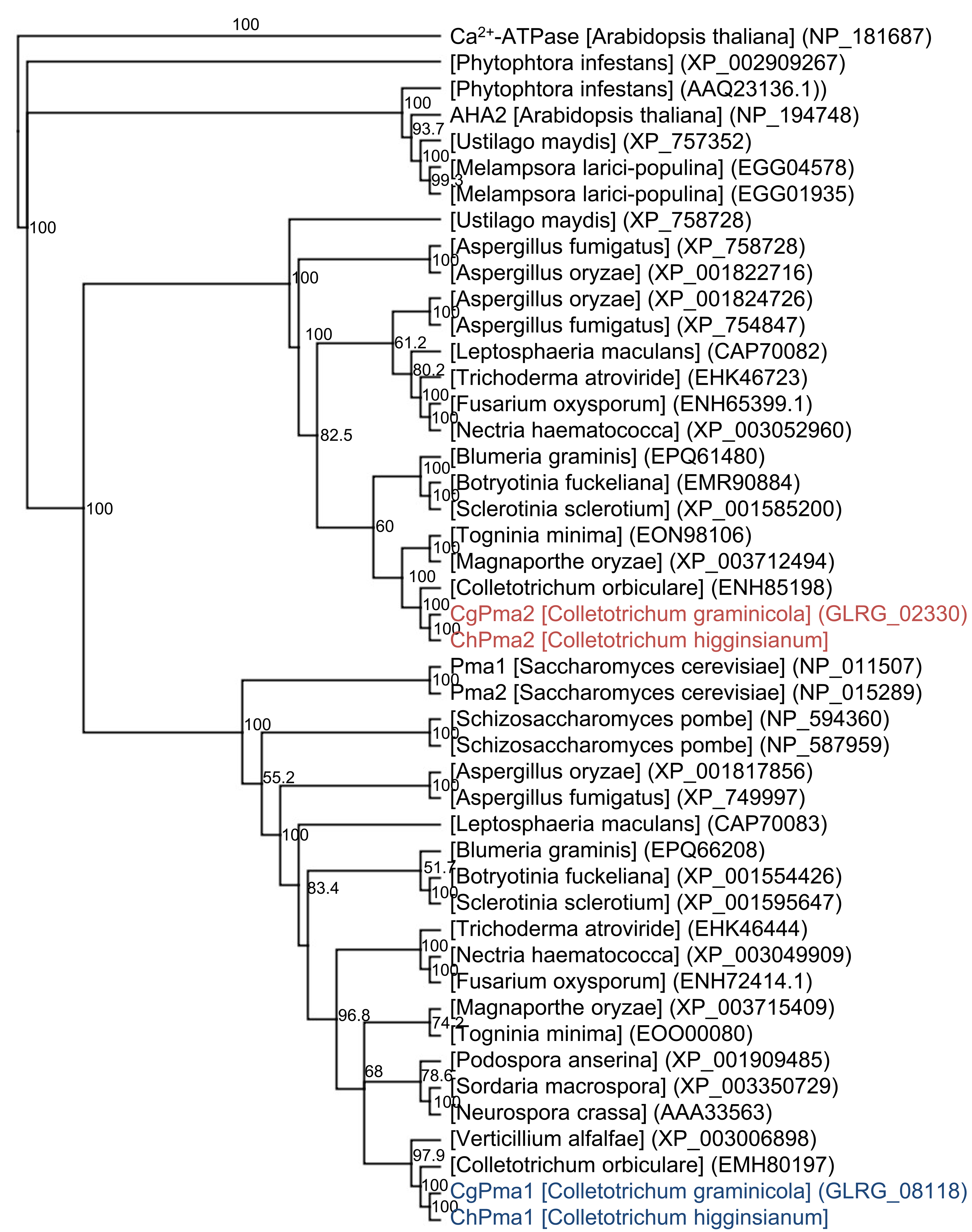

Supplement: S6 Fig — Phylogenetic analysis of fungal plasma membrane H+-ATPases. Sequences were aligned with ClustalW and the tree was generated by Geneious treebuilding (Jukes-Cantor; Neighbor-joining) with Ca2+ ATPase 4 from A. thaliana (NP_181687) as outgroup. Bootstrap values (1000 replicates) are indicated as percentage at the right side of the nodes. (TIF) [file pone.0125960.s006.tif]

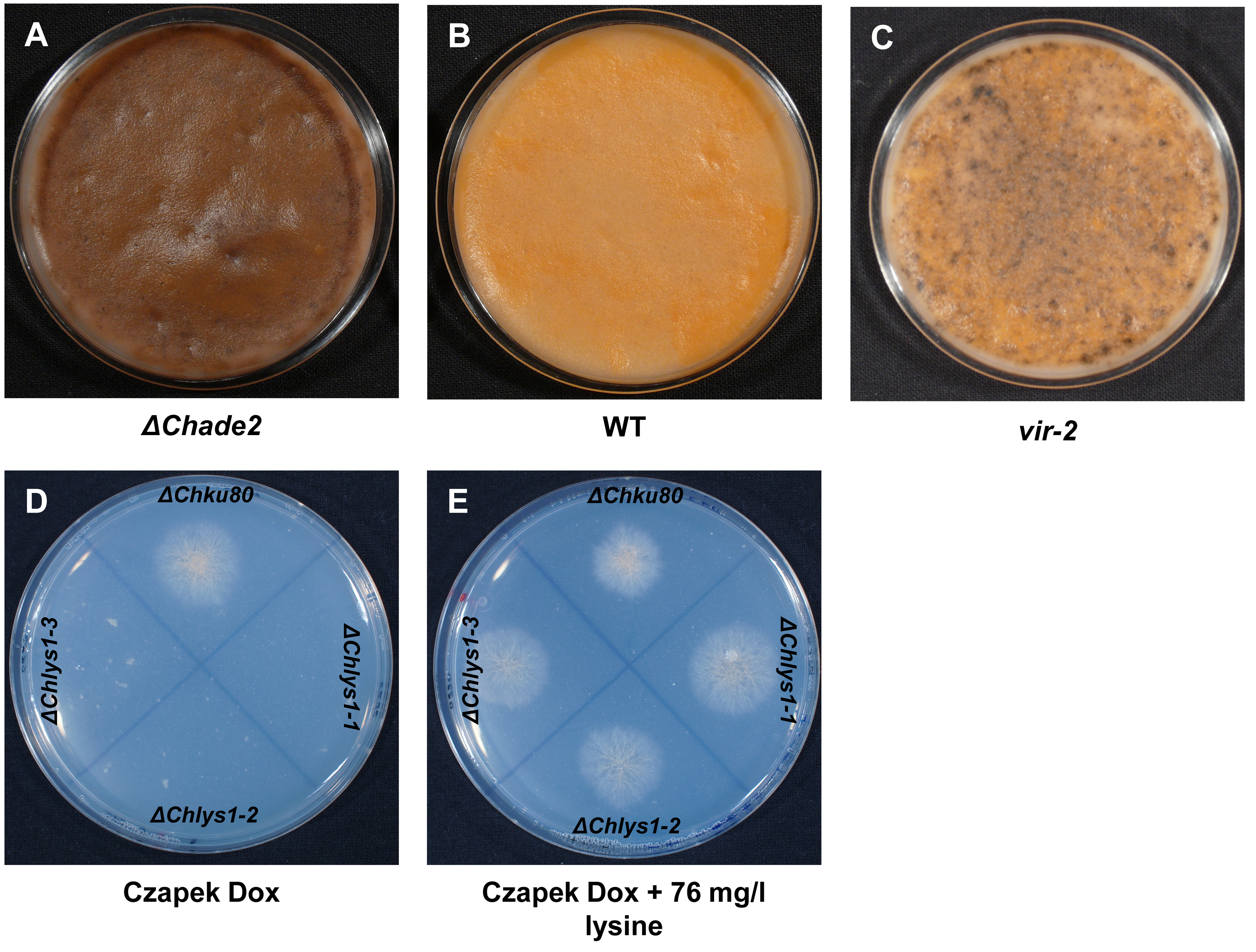

Supplement: S7 Fig — (A-C) Growth of C. higginsianum ΔChade2 (A), wildtype (B) and vir-2 strains (C) on oatmeal agar plates after seven days. (D-E) Growth of three C. higginsianum ΔChlys1 mutants and the ΔChku80 parental strain on Czapek Dox minimal medium (D) or on Czapek Dox supplemented with 76 mg/l lysine (E) after six days. (TIF) [file pone.0125960.s007.tif]

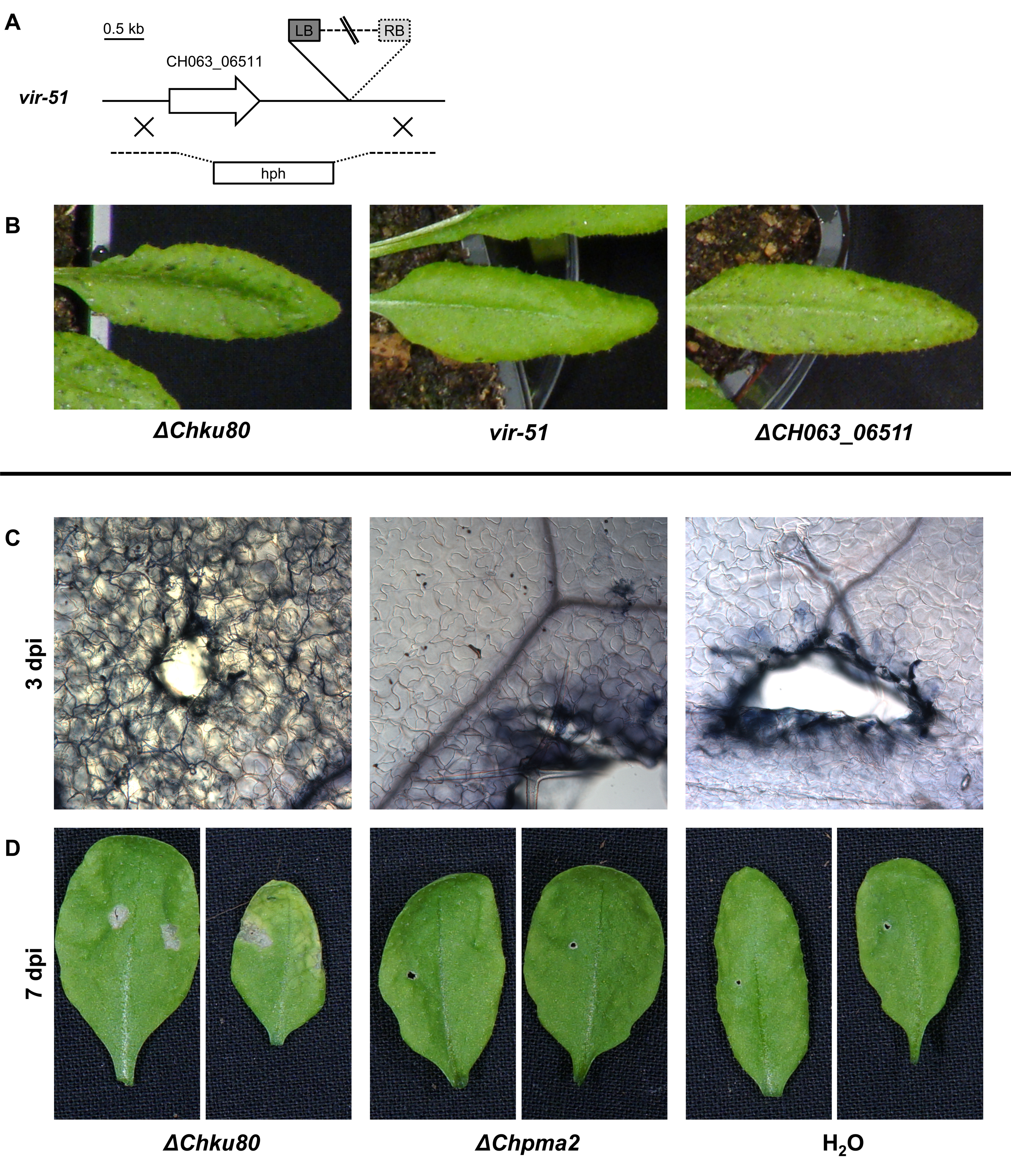

Supplement: S8 Fig — (A) Schematic representation of the T-DNA insertion site of vir-51. The homology regions used for targeted gene knockout by homologous recombination in the ΔChku80 strain are illustrated as dotted lines. The T-DNA border sequence identified by Genome Walker PCR is illustrated in dark gray. (B) A. thaliana plants four days after spray infection with ΔChku80, vir-51 and ΔCH063_06511 strains. (C) Wounded A. thaliana leaves 3 days after droplet inoculation with ΔChku80, ΔChpma2 or mock control (H2O). The area around the wounding site is shown after trypan blue staining. (D) Wounded A. thaliana leaves 7 days after droplet infection. The left side of each leaf was perforated with a single hole using a small pin, while the right side was left unwounded. (TIF) [file pone.0125960.s008.tif]
