# Supplementary material for: A Genetic Screen for Pathogenicity Genes in the Hemibiotrophic Fungus Colletotrichum higginsianum Identifies the Plasma Membrane Proton Pump Pma2 Required for Host Penetration
Source: PLoS One. 2015 May 19;10(5):e0125960. doi: 10.1371/journal.pone.0125960 (PMC4437780; doi:10.1371/journal.pone.0125960)
Supplement: S1 Text — Contains details about plasmid constructions, DNA primers and additional methods. (DOCX) [file pone.0125960.s009.docx]

**S1 Text. Supporting materials and methods**

**Genomic DNA preparation**

Genomic DNA was prepared from mycelium grown in 100 ml modified Mathur’s medium for 2 days at 28°C as described (Thon et al., 2000) with the following modifications: chloroform extraction was followed by an additional extraction with phenol and purified DNA was stored in 0.1 x TE buffer at 4°C. For isolation of total DNA from conidia, densely grown OMA plates were rinsed with sterile water to collect conidia. Conidia were washed twice in approximately 5 ml H_2_O. 4 x 10^8^ conidia/ml were resuspended in 200 µl SCE-mix (3 mg/ml zymolyase T20, 1 M sorbitol, 100 mM sodium citrate, pH 7.0, 60 mM EDTA, 100 mM 2-mercaptoethanol) and incubated for at least 60 minutes at 37°C until spheroplasts became visible under the microscope. 200 µl SDS-solution (2% SDS; 50 mM EDTA, 100 mM Tris-HCl, pH 9.0) was added, the suspension gently mixed and heated for 10 minutes at 75°C. For protein precipitation, 200 µl potassium acetate solution (5 M) was added, the suspension mixed, cooled for 20 minutes at 4°C and centrifuged for 30 minutes at 13000 rpm. DNA was precipitated from the supernatant with 200 µl ammonium acetate solution (2 M) and 1 ml isopropanol. DNA was resuspended in 100 µl 0.1 x TE buffer.

**RNA extraction**

Total RNA was extracted using the RiboPure^TM^-Yeast Kit (Ambion #AM1926). For RNA preparation from mycelium, 100 mg mycelium grown in modified, liquid Mathur’s medium was finely grounded in a mortar containing liquid nitrogen. Mycelial powder was transferred to small screw cap tubes and thawed by the addition of 1 ml lysis reagent (480 µl lysis buffer, 48 µl 10% SDS and 480 µl equilibrated phenol:chloroform:isoamyl alcohol (25:24:1) mixture) and 750 µl zirconia beads. For mechanical disruption, samples were treated for 10 minutes at 4°C at full speed on a vibrax shaker. After centrifugation, RNA was purified from the aqueous phase with glass-fiber filter cartridges according to the manufacturer’s instructions. Contaminating DNA was removed by DNaseI treatment with reagents provided by the Kit. RNA samples were stored at -80°C until use. RNA concentration was determined photometrically using a NanoDrop^®^ photometer (Peqlab, ND-1000). Preparation of conidial RNA was done from approximately 1-3 x 10^8^ conidia. Conidia were washed off from OMA plates, washed with water and the resulting conidial pellet was resuspended in 1 ml lysis reagent and processed as above. RNA from *in vitro* appressoria was prepared from 4 x 10^7^ appressoria formed on 1,16-hexadecanediol coated petri dishes overlaid with H_2_O. Appressoria (2 x 10^7^ appressoria/plate) were scraped off using a plastic inoculation spreader from four petri dishes (145 mm diameter) (Sarstedt, Germany) with 0.5 ml lysis reagent per plate. For cell lysis, the resulting suspension was added to zirconia beads (750 µl/tube with 1 ml cell lysate each). The combined lysates from 2 tubes were purified per cartridge.

**Identification of T-DNA insertion sites by Genome walker PCR**

Isolation of genomic sequences flanking T-DNA inserts (Siebert et al., 1995) was performed by PCR as described in the GenomeWalker™ Universal Kit User Manual (Clontech Laboratories, USA) with following modifications: 1 µg of genomic *C. higginsianum* DNA was digested overnight with either of the blunt end generating restriction endonucleases EcoRV, NaeI and PvuII (50 U). Digested DNA was extracted twice with 1 volume of phenol/chloroform/isoamyl alcohol (Carl Roth, Karlsruhe, Germany) and after adding 20 µg glycogen and 0.1 volume of 3 M sodium acetate, precipitated with 2 volumes of 100% ethanol. The DNA adaptor was prepared by phosphorylation of 12.5 µl oligonucleotide CK2581 (100 µM) with 10 units of T4 polynucleotide kinase (New England Biolabs, USA) for one hour at 37°C in 37.5 µl ligase buffer (New England Biolabs, USA). After heat-inactivation at 65°C, 12.5 µl of complementary oligonucleotide CK2580 (100 µM) was added and annealed at 25°C after heating to 95°C for five minutes. For adaptor ligation, 2.6 µl adaptor (25 µM) was ligated to 4 µl digested and purified DNA using 3 U T4 DNA Ligase. 1 µl adaptor-ligated DNA (diluted 1:10) was used as template in a primary PCR (50 µl) using adaptor-primer CK2582 together with primer CK2611 (specific for left border (LB) sequences in pPK2) or with primer CK2710 (specific for right border (RB) sequences) at 200 nM each and 5 U Taq Polymerase (30 cycles). Primary reactions were diluted 1:50 and used as template in a nested PCR using adaptor primer 2 (CK2583) together with CK2709 (LB-specific oligo) or CK2575 (RB-specific oligo) as above. Reaction products were analyzed on a agarose gels and either sequenced directly using adaptor primer 2 (CK2583) or after sub-cloning into pJet2.1 (CloneJet^TM^ PCR Cloning Kit, Thermo Fisher Scientific) or pGEM^®^-T Easy (pGEM^®^-T Easy Vector system, Promega, USA).

**Histochemical staining**

Quantification of infection structures and development of *C. higginsianum* was performed on whole leave mounts stained with lactophenol-trypan blue (10 mg trypan blue, 10 g phenol, 10 ml lactic acid, 10 ml deionized water) (Keogh et al., 1980). Leaves were boiled for 60 seconds in staining solution and decolorized using chloral hydrate (2.5 g chloral hydrate dissolved in 1 ml deionized water) for at least 24 hours. Destained leaves were mounted in deionized water and viewed using bright-field or phase-contrast microscopy. At least 100 appressoria and the corresponding infection structures were counted. Detection of reactive oxygen species was performed using DAB staining. Single leaves of spray-infected (1 x 10^6^ conidia/ml) *A. thaliana* plants were harvested after three days and stained over night in freshly prepared DAB solution (10 mg 3,3’-diaminobenzidine was dissolved in 1 ml HCl (1 mM) and subsequently diluted tenfold in 100 mM potassium phosphate buffer, pH 5.8) and used as described (Thordal-Christensen et al., 1997). The staining solution was removed after 12 h and the leaves were boiled in 99% ethanol for 10 minutes. Leaves were mounted with water and viewed with bright-field microscopy. Quantification of DAB staining was based on the ratio of DAB stained *A. thaliana* epidermis cells to total appressoria count. Histochemical staining of callose deposits with aniline blue was performed as described (Fernandez and Heath, 1986; Shimada et al., 2006). Infected leaves were harvested three days post infection and decolorized in 99% ethanol. Ethanol was replaced after 12 h with aniline blue staining solution (0.005% (w/v) aniline blue in 0.07 M sodium phosphate buffer, pH 9.0) and incubated for 1 h. The samples were washed three times with deionized water and observed with epifluorescence (Leica UV filter cube A; BP 340-380 nm, LP 425 nm). Quantification of aniline blue staining is based on the ratio of callose deposition beneath appressoria to total appressoria count.

**Infection of wounded leaves**

Infection of wounded leaves was performed as described (Huser et al., 2009) on 5 week old *A. thaliana* plants.

**Plasmid constructions**

Construction of a nourseothricin resistance cassette:

Plasmid pCK2321 is a YEplac181 derivative containing a 4 kb region encompassing the *ChTRPC* gene and was isolated as a clone complementing the *trp1-1* mutant of *S. cerevisiae*. Plasmid pSL-TrpC-nat (pCK2651) encoding the nourseothricin resistance from *Streptomyces noursei* under control of the *C. higginsianum* *TRPC* promoter was constructed by first cloning the *nat* coding region from pMF1-N (Brachmann et al., 2004) as an NdeI-EcoRV fragment in pSL1180 (Pharmacia) generating pCK2653. Subsequently the *ChTRPC* promoter region from position -811 to -3 from the AUG was amplified with primer CK2621 and CK2571 with pCK2321 as template and inserted as NotI-NdeI fragment into NotI and NdeI digested pCK2653. Finally, a PCR fragment (primer CK2623 and CK2622) containing the 3´-UTR region of *ChTRPC* (position +2300 to +3139 counted from the AUG in CH063_10922) was inserted into EcoRV and BamHI of the resulting plasmid to generate pSL-TrpC-nat (pCK2651).

Construction of the binary vector pPN harboring a nourseothricin resistance cassette:

The hygromycin resistance cassette of pPK2 (Covert et al., 2001) was removed by EcoRV–HindIII digestion and was subsequently replaced by an EcoRV–HindIII fragment containing the multiple cloning region from pSL1180 yielding pCK2367. A BamHI-NotI fragment from pSL-TrpC-nat (pCK2651) containing the nourseothricin resistance cassette was filled in using T4 DNA polymerase and cloned into the EcoRV site of pCK2367 generating pPN (pCK2650).

Construction of a *ChKU80* deletion plasmid:

Plasmid pSL-TrpC-nat (pCK2651) was used to generate a *ChKU80* deletion construct. First, an 1163 bp XhoI-SalI fragment (generated by primer CK2689 and CK2690) containing *ChKU80* (CH063_02085) upstream sequences (-1132 to +31) was cloned into the XhoI site of pSL-TrpC-nat. An 1166 bp fragment of the 3´-UTR of *ChKU80* (+2353 to +3518), amplified using primer CK2691 and CK2692, was cloned into BamHI, generating pCK2797. Finally, pCK2797 was digested by XhoI and the resulting fragment containing both *ChKU80* flanking regions and the nat resistance cassette was cloned into the unique PmeI site of the T-DNA of pPK2 (Covert et al., 2001) after filling in 5’-overhangs with Klenow Fragment to generate pCK2831.

Generation of deletion strains using the pOSCAR system:

Plasmids for targeted gene replacements with the hph resistance cassette were constructed using the pOSCAR-System (Paz et al., 2011). Sequences flanking the target regions (see section below) on either side were amplified by PCR using primer pairs containing attachment sites at their 5´-end. 10 ng of each PCR fragment was used in a 5 µl BP-reaction together with 60 ng pA-Hyg-OSCAR (GenBank Accession no. HM623915), 60 ng pOSCAR (GenBank Accession no. HM623914) and 1 µl BP clonase® II enzyme mix (Invitrogen). The reaction was incubated at 25°C for 16 h and stopped by adding 0.5 µl proteinase K (20 µg/µl) for 10 minutes at 37°C. 2 µl of this reaction mixture was used to transform competent *E. coli* cells. Colonies were selected with 100 µg/ml spectinomycin and clones were verified using multiple restriction enzyme digestions. *A. tumefaciens* strain AGL1 transformed with the respective replacement constructs was used for *C. higginsianum* transformation. Hygromycin resistant transformants were analyzed for successful homologous recombination by analyzing their chromosomal DNA by PCR. Absence of targeted sequences was tested by using primer from the coding region, while homologous integration of the construct at the desired genomic locus was confirmed by using PCR primer binding to chromosomal DNA sequences flanking the regions present on the targeting constructs.

Plasmids for targeted gene replacement:

5´ and 3´ flanking sequences of the following genes were used to generate knockout plasmids. A 972 bp fragment containing the 5´- flanking region of *ChPMA2* (-386 to +586) and a 849 bp fragment containing the 3´- flanking region (+2803 to +3652) was amplified using primer CK3319 together with CK3320 and CK3321 together with CK3329, respectively. The resulting *ChPMA2* knockout plasmid pDelPMA2 (pCK3349) deletes most of the coding region (corresponding to amino acids 156 – 873 in ChPma2 (1016 aa total)).

The *ChPMA1* knockout plasmid pCK3495 was constructed in an analogous manner using an 819 bp 5’ flanking region (-318 to +501; primer CK3383 and CK3384) and a 900 bp 3’ flanking fragment (+2787 to +3687; primer CK3385 and CK3386) for the BP clonase reaction.

pCK3822 (deletion of CH063_06511 and approximately 2000 bp of downstream sequence) was constructed using a 762 bp 5’ flanking region (-684 to +81; primer CK3660 and CK3661) and a 776 bp 3’ flanking region (+2313 to +3088; primer CK3668 and CK3669), pCK3650 (deletion of *ChLYS1*) using a 845 bp 5’ flanking region (-760 to +65; primer CK3611 and CK3612) and a 756 bp 3’ flanking region (+892 to +1650; primer CK3609 and CK3610), pCK3475 (deletion of *ChADE2*) using a 1026 bp 5’ flanking region (-1001 to +25; primer CK3355 and CK3356) and a 907 bp 3’ flanking region (+1991 to +2898; primer CK3357 and CK3358) and pCK3500 (deletion of *ChSTE12*) using a 1014 bp 5’ flanking region (-622 to +392; primer CK3419 and CK3420) and a 906 bp 3’ flanking region (+2226 to +3131; primer CK3421 and CK3422). All constructs were tested by restriction analysis and introduced into the *ΔChku80-1* strain (CY6021) by ATMT.

*ChPMA2* promoter mCherry fusion construct (Ppma2-mCherry):

A PCR fragment encompassing the *ChPMA2* promoter (-1042 to -1) was amplified using primer CK3529 and CK3530 and cloned as EcoRV-NdeI fragment into pSL1180, yielding pCK3538. mCherry was amplified from pm-rb (Nelson et al., 2007) using primer CK3870 and CK3871 and subsequently cloned as NdeI-NotI fragment into pCK3538, resulting in construct pCK3882. pCK3882 was digested by EcoRI and NotI, the fragment containing the *ChPMA2* promoter and mCherry was filled in with T4 DNA Polymerase and cloned into PmeI of pPN creating Ppma2-mCherry (pCK3880).

*ChPMA1* promoter GFP fusion construct (Ppma1-GFP):

A 200 bp PCR fragment containing the terminator region with a putative polyA-site of the *C. higginsianum* pyruvate kinase gene (CH063_11345) was amplified using CK3706 and CK3728 and cloned as EcoRV-PmeI fragment into the PmeI site of pPK2 yielding pCK3769. The PmeI site of pCK3769 was used to insert a sGFP fragment amplified from pMF280 (Freitag et al., 2004) with primer CK3709 and CK3747 as a NaeI-PmeI fragment, resulting in pCK3786. A 641 bp *ChPMA1* promoter fragment (primer CK3928 and CK3930) was integrated as an EcoRV fragment into the PmeI site of pCK3786 generating Ppma1-GFP (pCK3973).

Additional vectors used for T-DNA insertion mutagenesis:

*C. higginsianum* transformants generated for our mutant screen by ATMT were created using pPK2 (Covert et al., 2001) with the exception of those listed below, which were generated using the following unpublished pPK2 derivatives: pCK2508, pCK2329, pCK2391, pCK2531, pCK2432, pCK2448, and pCK2450. pCK2448 encodes GFP and was generated by digestion of p123 (Basse et al., 2000) with NotI and EcoRV and ligation with a NotI-EcoRV digested PCR fragment from the *ChTRPC* terminator (primers CK2400 and CK2401) resulting in pCK2426. pCK2426 was digested with NcoI and EcoRV generating a GFP-TrpC terminator fragment which was ligated together with a PCR fragment of the promoter region of *ChTRPC* (primer CK2322 and CK2407, PCR product digested with NcoI and EcoRV) into the EcoRV site of pPK2. pCK2450 was created similarly by integrating the GFP cassette into the single PmeI site of pPK2.

**Plasmids used for generation of random insertional mutants.**

| mutant | plasmid used for transformation |
| --- | --- |
| *vir-2* | pCK2450 |
| *vir-10* | pCK2448 |
| *vir-11* | pCK2448 |
| *vir-12* | pCK2450 |
| *vir-15* | pCK2508 |
| *vir-40* | pCK2329 |
| *vir-41* | pCK2391 |
| *vir-42* | pCK2391 |
| *vir-43* | pCK2508 |
| *vir-44* | pCK2508 |
| *vir-45* | pCK2450 |
| *vir-70* | pCK2450 |
| *vir-71* | pCK2531 + pCK2432 |
| *vir-73* | pCK2391 |
| *vir-74* | pCK2448 |
| *vir-77* | pCK2450 |
| *vir-79* | pCK2508 |
| all other *vir* mutants | pPK2 |

**Additional plasmids used in this study**

| plasmid | important features | application | reference |
| --- | --- | --- | --- |
| pPK2 | hph resistance | cloning vector, used for ATMT mutagenesis of *C. higginsianum* | Covert et al., 2001 |
| pPN (pCK2650) | pPK2 derivate, where hph is replaced by nat | cloning vector, used for ATMT of *C. higginsianum* | this study |
| pm-rb | mCherry | source of mCherry ORF | Nelson et al., 2007 |
| pA-Hyg-OSCAR | hygromycin, attachment sites (P1r and P4) for recombination with pOSCAR | generation of knock out and/or deletion constructs | Paz et al., 2011 |
| pOSCAR | ccdB gene, attachment sites P2r and P3, RB and LB | generation of knock out and/or deletion constructs | Paz et al., 2011 |
| pSL1180 | multiple cloning region | cloning vector | Amersham |
| pMF1-N | nourseothricin (nat) ORF | source for nourseothricin ORF | Brachmann et al., 2004 |
| pMF280 | sGFP | Source of sGFP ORF | Freitag et. al., 2004 |
| pCK2321 | YEPlac181+4 kb genomic region of *ChTRPC* | complementation of *S. cerevisiae* tryptophan mutants | unpublished results |
| pCK2329 | pPK2 derivative (GFP) | used for ATMT mutagenesis of *C. higginsianum* | this study |
| pCK2391 | pPK2 derivative (GFP) | *C. higginsianum* promoter trap vector, used for ATMT mutagenesis of *C. higginsianum* | this study |
| pCK2432 | pPK2 derivative (CFP) | used for ATMT mutagenesis of *C. higginsianum* | this study |
| pCK2448 | pPK2 derivative (GFP) | used for ATMT mutagenesis of *C. higginsianum* | this study |
| pCK2450 | pPK2 derivative (GFP) | used for ATMT mutagenesis of *C. higginsianum* | this study |
| pCK2508 | pPK2 derivate (promoter less thymidine kinase of HSV) | used for ATMT mutagenesis of *C. higginsianum* | this study |
| pCK2651  pSL-TrpC-nat | pSL1180 derivative (nat ORF) | source of PtrpC-nat | this study |
| pCK2831 | pPN derivative | knock out of *ChKU80* | this study |
| pCK3349 | pOSCAR derivative | knock out of *ChPMA2* | this study |
| pCK3475 | pOSCAR derivative | knock out of *ChADE2* | this study |
| pCK3495 | pOSCAR derivative | knock out of *ChPMA1* | this study |
| pCK3500 | pOSCAR derivative | knock out of *ChSTE12* | this study |
| pCK3650 | pPK2 derivative | knock out of *ChLYS1* | this study |
| pCK3822 | pOSCAR derivative | Knock out of *CH063_06511* | this study |
| pCK3880 | pPN derivative | Ppma2-mCherry | this study |
| pCK3882 | pSL1180 derivative | Ppma2-mCherry | this study |
| pCK3973 | pPK2 derivative | Ppma1-GFP | this study |

**DNA oligonucleotide primers used in this study**

| oligo | DNA sequence | Description/use |
| --- | --- | --- |
| CK2322 | 5’-ACGATATCATCTTGAAGAAACTGTACAAC | forward primer for PtrpC (EcoRV) |
| CK2400 | 5’-GGGCGGCCGCAAGGTATTAGCTATTTATTCAAGG | forward primer for TtrpC (NotI) |
| CK2401 | 5’-GGATATCGCATTAAGCACAGAGATATGCTTG | reverse primer for TtrpC (EcoRV) |
| CK2407 | 5’-CGCCATGGTGGATTCTTGGTGCTGCAACCACC | reverse primer for PtrpC (NcoI) |
| CK2571 | 5’-ACGGCATATgGGATTCTtGGTGCTGCAACCACC | reverse primer for PtrpC (NdeI) |
| CK2575 | 5’-CCTGAATGGCGAATGAGCTTGAGCTT | pPK2 right border nested primer |
| CK2580 | 5’-GTAATACGACTCACTATAGGGCACGCGTGGTCGACGGCCCGGGCTGGT | oligo 2 for linker adaptor synthesis |
| CK2581 | 5’-ACCAGCCC-NH2 | oligo 1 for linker adaptor synthesis with 3’-end amino-C7 linker |
| CK2582 | 5’-GTAATACGACTCACTATAGGGC | genome walker adaptor primer 1 |
| CK2583 | 5’-ACTATAGGGCACGCGTGGT | genome walker adaptor primer 2 |
| CK2611 | 5’-AATCGGCCAACGCGCGGGGAGAGGCGGTTTG | pPK2 left border primer |
| CK2621 | 5’-GGGCGGCCGCATCATCTTGAAGAAACTGTACA | forward primer for PtrpC (NotI) |
| CK2622 | 5’-TAGGATCCTTAAGCACAGAGATATGCTTGTGT | reverse primer for TtrpC (BamHI) |
| CK2623 | 5’-TCGATATCCAAGGTATTAGCTATTTATTCAAG | forward primer for TtrpC (EcoRV) |
| CK2689 | 5’-ctggatggtgaagtagacggg | forward primer for *ChKU80* upstream homology region |
| CK2690 | 5’-gaccgtggtcgatatcttgt | reverse primer for *ChKU80* upstream homology region |
| CK2691 | 5’-aatctaggatCCtcaaggggaagatcctctc | forward primer for KU80 downstream homology region (BamHI) |
| CK2692 | 5’-ttgtagggatCCGCTCTGGATAAGCCGTTGC | reverse primer for KU80 downstream homology region (BamHI) |
| CK2709 | 5’-ATTCGGCGTTAATTCAGTACATTAAAAACG | pPK2 left border nested primer |
| CK2710 | 5’-TAATAGCGAAGAGGCCCGCACCGATCGC | pPK2 right border nested primer |
| CK3319 | 5’-GGGGACAGCTTTCTTGTACAAAGTGGAACTCCATTGCCGCGCTGTACTTCG | attB2r for deletion of *ChPMA2* |
| CK3320 | 5’-GGGGACTGCTTTTTTGTACAAACTTGTGAATGGGACCACGGAAGAAACCGA | attB1r for deletion of *ChPMA2* |
| CK3321 | 5’-GGGGACAACTTTGTATAGAAAAGTTGTTTCCATCGTCCTCGGTGGTCTTCTC | attB4 for deletion of *ChPMA2* |
| CK3329 | 5’-GGGGACAACTTTGTATAATAAAGTTGTCATTAGTGACAGTCGTATGATGTCGC | attB3r for deletion of *ChPMA2* |
| CK3355 | 5'-GGGGACAGCTTTCTTGTACAAAGTGGAATGTGGGGAGGGAAGAGTACGGCAA | attB2r for deletion of *ChADE2* |
| CK3356 | 5'-GGGGACTGCTTTTTTGTACAAACTTGTGGCCGATGATGGGCTTCTTGACCA | attB1r for deletion of *ChADE2* |
| CK3357 | 5'-GGGGACAACTTTGTATAGAAAAGTTGTTTACCAGCAACGGCGTACACAAGGG | attB4 for deletion of *ChADE2* |
| CK3358 | 5'-GGGGACAACTTTGTATAATAAAGTTGTTCCTGGGACACAAGGTGGACCTGA | attB3 for deletion of *ChADE2* |
| CK3383 | 5’-GGGGACAGCTTTCTTGTACAAAGTGGAAACCAAACTTTCCTCTCCCTCC | attB2r for deletion of *ChPMA1* |
| CK3384 | 5’-GGGGACTGCTTTTTTGTACAAACTTGTACGAACTGGATAGGACCAACG | attB1r for deletion of *ChPMA1* |
| CK3385 | 5’-GGGGACAACTTTGTATAGAAAAGTTGTTATCTGGATCTTCTCCTTCGGC | attB4 for deletion of *ChPMA1* |
| CK3386 | 5´-GGGGACAACTTTGTATAATAAAGTTGTCATCTCGAGAACCCCTACTGG | attB3r for deletion of *ChPMA1* |
| CK3419 | 5’-GGGGACAGCTTTCTTGTACAAAGTGGAACCATCATCGCGAATCTCATCGCG | attB2r for deletion of *ChSTE12* |
| CK3420 | 5’-GGGGACTGCTTTTTTGTACAAACTTGTGATGCCCTCCTCGAACTTCTTGG | attB1r for deletion of *ChSTE12* |
| CK3421 | 5’-GGGGACAACTTTGTATAGAAAAGTTGTTTGGCGAGAACGGCGAGGCTGC | attB3 for deletion of *ChSTE12* |
| CK3422 | 5’-GGGGACAACTTTGTATAATAAAGTTGTTTGCCGAACATTCTCTCCTACCC | attB4 for deletion of *ChSTE12* |
| CK3529 | 5’-GCTGAGAGACTTGACATTTACCCC | forward primer for Ppma2 |
| CK3530 | 5’-TTGGCATATGAAACTGTCGGAAAAGGATTACAGTCC | reverse primer for Ppma2 (NdeI) |
| CK3609 | 5’-GGGGACAGCTTTCTTGTACAAAGTGGAAACGGCTTGGGACACCCTGCTC | attB2r for deletion of *ChLYS1* |
| CK3610 | 5’-GGGGACTGCTTTTTTGTACAAACTTGTCCGCAGACACCACCAACCC | attb1r for deletion of *ChLYS1* |
| CK3611 | 5’-GGGGACAACTTTGTATAGAAAAGTTGTTCCGAGAGTCTTCTCGAAACCACC | attB4 for deletion of *ChLYS1* |
| CK3612 | 5’-GGGGACAACTTTGTATAATAAAGTTGTGCACCTGAGTATCTACTCTTGGC | attB3 for deletion of *ChLYS1* |
| CK3660 | 5’-GGGGACAGCTTTCTTGTACAAAGTGGAAGCATGTTCTGCTGATGTTCTGAG | attB2r for deletion of CH063_06511 |
| CK3661 | 5’-GGGGACTGCTTTTTTGTACAAACTTGTCTTCTGGTCGTCGCAGGCGAT | attB1r for deletion of CH063_06511 |
| CK3668 | 5’-GGGGACAACTTTGTATAGAAAAGTTGTTTGCAGCGCGAAATAGTCACA | attB4 for deletion of CH063_06511 |
| CK3669 | 5’-GGGGACAACTTTGTATAATAAAGTTGTACCGACGCTGCGGAAGTTGG | attB3 for deletion of CH063_06511 |
| CK3747 | 5’-AATGCCGGCTTACTTGTACAGCTCGTCCATGC | reverse GFP primer (NaeI) |
| CK3870 | 5’-aacccatATGGTGAGCAAGGGCGAGGA | forward mCherry primer (NdeI) |
| CK3871 | 5’-taagcggccgcTTACTTGTACAGCTCGTCCATG | reverse mCherry primer (NotI) |
| CK3928 | 5’-aaaaGATATCCACTTGCAGTCAATCGCGCC | forward primer for Ppma1 (EcoRV) |
| CK3930 | 5’-aaaaGATATCTGCGGTGGTTGCGAGACAGA | reverse primer for Ppma1 (EcoRV) |
| CK3959 | 5'-ACAACGAGGCCATCTACGA | forward qRT-PCR primer for α-tubulin |
| CK3960 | 5'-GGAGGAAACGACCTGAGCA | reverse qRT-PCR primer for α-tubulin |
| CK4279 | 5-CGCCGGTGTCTACTACCTCCTT | forward qRT-PCR primer for *ChPMA1* |
| CK4280 | 5-CGCTGCATAGAGACGACGAAG | reverse qRT-PCR primer for *ChPMA1* |
| CK4281 | 5-CTTTGCTTGCACTTGGAGATCTAC | forward qRT-PCR primer for *ChPMA2* |
| CK4282 | 5-GTGAGCGTTGTCGTAGGCAA | reverse qRT-PCR primer for *ChPMA2* |

**References for S1 Text**

Basse, C.W., Stumpferl, S., and Kahmann, R. (2000). Characterization of a *Ustilago maydis* Gene Specifically Induced during the Biotrophic Phase: Evidence for Negative as Well as Positive Regulation. Mol. Cell. Biol. *20*, 329–339.

Brachmann, A., König, J., Julius, C., and Feldbrügge, M. (2004). A reverse genetic approach for generating gene replacement mutants in *Ustilago maydis*. Mol. Genet. Genomics *272*, 216–226.

Covert, S.F., Kapoor, P., Lee, M., Briley, A., and Nairn, C.J. (2001). *Agrobacterium tumefaciens*-mediated transformation of *Fusarium circinatum*. Mycol. Res. *105*, 259–264.

Fernandez, M.R., and Heath, M.C. (1986). Cytological responses induced by five phytopathogenic fungi in a nonhost plant, *Phaseolus vulgaris*. Can. J. Bot. *64*, 648–657.

Freitag, M., Hickey, P.C., Raju, N.B., Selker, E.U., and Read, N.D. (2004). GFP as a tool to analyze the organization, dynamics and function of nuclei and microtubules in *Neurospora crassa*. Fungal Genet. Biol. *41*, 897–910.

Huser, A., Takahara, H., Schmalenbach, W., and O’Connell, R. (2009). Discovery of pathogenicity genes in the crucifer anthracnose fungus *Colletotrichum higginsianum*, using random insertional mutagenesis. Mol. Plant. Microbe. Interact. *22*, 143–156.

Keogh, R.C., Deverall, B.J., and McLeod, S. (1980). Comparison of histological and physiological responses to *Phakopsora pachyrhizi* in resistant and susceptible soybean. Trans. Br. Mycol. Soc. *74*, 329–333.

Nelson, B.K., Cai, X., and Nebenführ, A. (2007). A multicolored set of in vivo organelle markers for co-localization studies in *Arabidopsis* and other plants. Plant J. *51*, 1126–1136.

Paz, Z., García-Pedrajas, M.D., Andrews, D.L., Klosterman, S.J., Baeza-Montañez, L., and Gold, S.E. (2011). One step construction of *Agrobacterium*-Recombination-ready-plasmids (OSCAR), an efficient and robust tool for ATMT based gene deletion construction in fungi. Fungal Genet. Biol. *48*, 677–684.

Shimada, C., Lipka, V., O’connell, R.J., Okuno, T., Schulze-lefert, P., and Takano, Y. (2006). Nonhost resistance in *Arabidopsis*-*Colletotrichum* interactions acts at the cell periphery and requires actin filament function. Mol. Plant Microbe Interact. *19*, 270–279.

Siebert, P.D., Chenchik, A., Kellogg, D.E., Lukyanov, K.A., and Lukyanov, S.A. (1995). An improved PCR method for walking in uncloned genomic DNA. Nucleic Acids Res. *23*, 1087–1088.

Thon, M.R., Nuckles, E.M., and Vaillancourt, L.J. (2000). Restriction enzyme-mediated integration used to produce pathogenicity mutants of *Colletotrichum graminicola*. Mol. Plant. Microbe. Interact. *13*, 1356–1365.

Thordal-Christensen, H., Zhang, Z., Wei, Y., and Collinge, D.B. (1997). Subcellular localization of H_2_O_2_ in plants. H_2_O_2_ accumulation in papillae and hypersensitive response during the barley-powdery mildew interaction. Plant J. *11*, 1187–1194.
